# AGC kinases and MAB4/MEL proteins maintain PIN polarity by limiting lateral diffusion in plant cells

**DOI:** 10.1016/j.cub.2021.02.028

**Published:** 2021-05-10

**Authors:** Matouš Glanc, Kasper Van Gelderen, Lukas Hoermayer, Shutang Tan, Satoshi Naramoto, Xixi Zhang, David Domjan, Ludmila Včelařová, Robert Hauschild, Alexander Johnson, Edward de Koning, Maritza van Dop, Eike Rademacher, Stef Janson, Xiaoyu Wei, Gergely Molnár, Matyáš Fendrych, Bert De Rybel, Remko Offringa, Jiří Friml

**Affiliations:** 1 Institute of Science and Technology Austria (IST Austria), 3400 Klosterneuburg, Austria; 2 Plant Developmental Genetics, Institute of Biology Leiden, Leiden University, 2333 BE Leiden, the Netherlands; 3 Department of Experimental Plant Biology, Faculty of Science, Charles University, 12844 Prague, Czechia; 4 Department of Plant Biotechnology and Bioinformatics, Ghent University, 9052 Ghent, Belgium; 5 VIB Center for Plant Systems Biology, 9052 Ghent, Belgium; 6 Plant Ecophysiology, Institute of Environmental Biology, Utrecht University, 3584 CH Utrecht, the Netherlands; 7 Department of Biological Sciences, Faculty of Science, Hokkaido University, Sapporo, Hokkaido 060-0810, Japan; 8 Department of Applied Genetics and Cell Biology, University of Natural Resources and Life Sciences, (BOKU), 1190 Vienna, Austria

**Keywords:** cell polarity, lateral diffusion, protein phosphorylation, polar auxin transport, *Arabidopsis*, plant development, positive feedback

## Abstract

Polar subcellular localization of the PIN exporters of the phytohormone auxin is a key determinant of directional, intercellular auxin transport and thus a central topic of both plant cell and developmental biology. *Arabidopsis* mutants lacking PID, a kinase that phosphorylates PINs, or the MAB4/MEL proteins of unknown molecular function display PIN polarity defects and phenocopy *pin* mutants, but mechanistic insights into how these factors convey PIN polarity are missing. Here, by combining protein biochemistry with quantitative live-cell imaging, we demonstrate that PINs, MAB4/MELs, and AGC kinases interact in the same complex at the plasma membrane. MAB4/MELs are recruited to the plasma membrane by the PINs and in concert with the AGC kinases maintain PIN polarity through limiting lateral diffusion-based escape of PINs from the polar domain. The PIN-MAB4/MEL-PID protein complex has self-reinforcing properties thanks to positive feedback between AGC kinase-mediated PIN phosphorylation and MAB4/MEL recruitment. We thus uncover the molecular mechanism by which AGC kinases and MAB4/MEL proteins regulate PIN localization and plant development.

## Highlights

• MAB4/MEL proteins are recruited to the plasma membrane by PINs
• PINs, MAB4/MELs, and AGC kinases directly interact in a multiprotein complex
• PIN phosphorylation and MAB4/MEL recruitment form a positive feedback loop
• MAB4/MELs and AGC kinases maintain PIN polarity by limiting PIN lateral diffusion

## Graphical abstract

**Figure:**
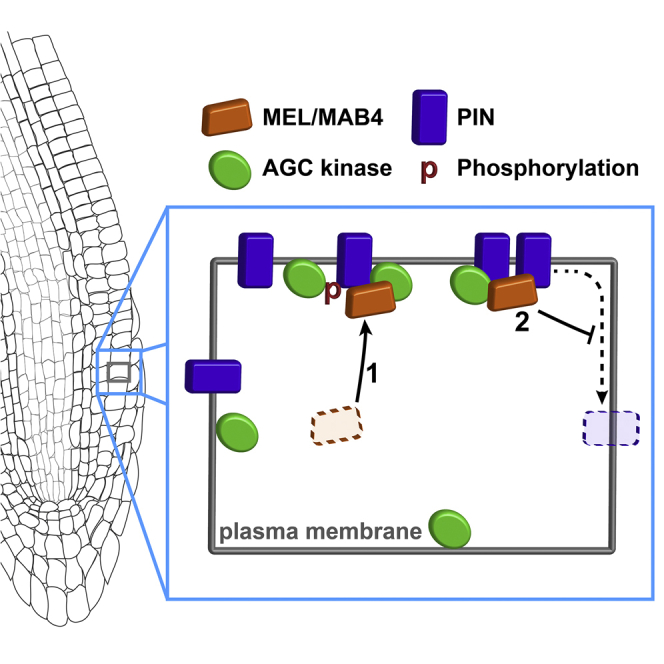

## Teaser

Glanc, Van Gelderen et al. show that PIN auxin transporters, AGC kinases, and MAB4/MEL proteins form a complex at the plasma membrane. This complex has self-reinforcing properties thanks to positive feedback between PIN phosphorylation and MAB4/MEL recruitment, and contributes to PIN polarity maintenance by limiting PIN lateral diffusion.

## Introduction

Auxin is a versatile regulator of plant growth and development. Plants perceive and integrate various internal and external stimuli into local auxin maxima and minima, which are translated into different developmental outputs. This asymmetric distribution of auxin is achieved mainly through polar auxin transport, which is in turn heavily dependent on the polar subcellular plasma membrane (PM) localization of the PIN-FORMED (PIN) auxin efflux carriers.^1^, ^2^, ^3^, ^4^, ^5^

Certain developmental processes, for example the maintenance of some stem cell niches, need auxin maxima to be remarkably stable over time,^6^^,^^7^ whereas others, such as organ initiation at the shoot apical meristem, wound healing, or photo- and gravitropic responses, rely on dynamic changes rather than stable patterns of auxin distribution.^8^, ^9^, ^10^, ^11^, ^12^ Hence, plant cells must possess mechanisms to maintain the underlying PIN polar localization over long periods and yet be able to change it quickly in response to miscellaneous signaling inputs. Despite PIN polar localization clearly being a crucial determinant of plant development,^3^ our knowledge of the underlying molecular mechanisms is still fragmented. In yeast, studies of the canonical Cdc42 polarity establishment pathway have established protein phosphorylation, specific lateral diffusion rates of different polarity proteins, and positive feedback as key mechanisms of symmetry breaking.^13^^,^^14^ Also, in the case of PIN polarity establishment and/or maintenance, a role of limited lateral diffusion has been suggested;^15^^,^^16^ however, its mechanistic basis is not understood.

One of the few established PIN polarity regulators is the AGC3 protein Ser/Thr kinase PINOID (PID) and its WAG1 and WAG2 homologs, which control polar localization of PINs by directly phosphorylating their central hydrophilic loop (HL).^17^, ^18^, ^19^, ^20^, ^21^, ^22^ These AGC3 kinases act in the same pathway with plant-specific proteins of unknown molecular function encoded by the *MAB4/MEL* (*MACCHI-BOU 4/MAB4(ENP1)-LIKE*) gene family.^23^, ^24^, ^25^, ^26^, ^27^ Different *pid/wag* and *mab4/mel* mutant combinations, in line with their PIN polarity defects, often phenocopy *pin* mutants: *pid* and *mab4* produce naked inflorescence stems similar to *pin1*, and *pid wag1 wag2* as well as *mel1234* have agravitropic roots reminiscent of *pin2*.^24^^,^^26^^,^^28^ The MAB4/MEL proteins harbor an N-terminal Broad-Complex, Tramtrack, and Bric-a-brac (BTB) and a C-terminal plant-specific NPH3 domain. BTB domains are well known for mediating protein-protein interactions in both plants and animals (Robert et al.^29^ and references therein). The NPH3 domain is named after NON-PHOTOTROPIC-HYPOCOTYL3, which is known as a signal transducer of blue-light-induced phototropism and cooperates with PID, WAG1, and WAG2 in regulating PIN3 polarity in the hypocotyl.^8^^,^^30^ Besides being reversibly phosphorylated, the molecular action of the NPH3 domain is unknown.^31^

The polarity of both PIN1-GFP and PIN2-GFP is significantly reduced in the *mel1234* mutant, and MAB4/MEL proteins localize to the same polar domains as PINs in all tissues examined so far.^26^^,^^27^ Nevertheless, the mechanism by which MAB4/MELs regulate PIN localization and whether MAB4/MEL polarity is instructive for PIN polarity, or vice versa, have not been resolved.^32^ Moreover, precisely how PIN polarity is instructed through phosphorylation by the apolarly localized PID/WAGs is also a matter of debate.^32^^,^^33^

Here we show that PINs, AGC kinases, and MAB4/MEL proteins form a polar protein complex, which reinforces polar PIN localization. PINs interact with and are phosphorylated by the AGC kinases, and recruit the soluble MAB4/MELs to the polar domain of the PM. The efficiency of MAB4/MEL recruitment is tightly correlated with PIN phosphorylation status, together forming a positive feedback of the PIN-MAB4/MEL-AGC kinase complex, which restricts lateral diffusion-based escape of PINs from their polar domains. Hence, PIN polarity maintenance in plants depends on phosphorylation, a protein-protein interaction positive feedback loop, and specific lateral diffusion rates of its components, analogous to the molecularly unrelated Cdc42-dependent symmetry-breaking pathway in yeast.

## Results

### PINs recruit MAB4/MELs to different polar domains at the PM

**Figure 1:**
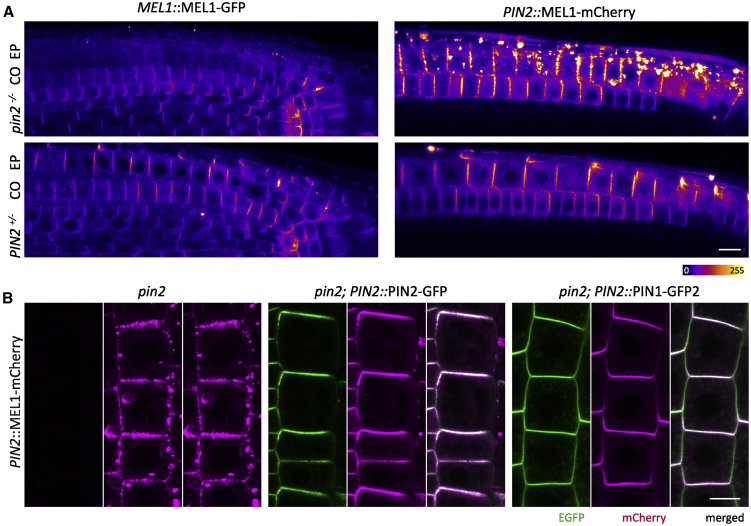
MEL1 is recruited to the PM by PINs *in planta* (A) Expression pattern and subcellular localization of *MEL1*::MEL1-GFP and *PIN2*::MEL1-mCherry in the *eir1-1 (pin2)* background and after a backcross with the WT (Col-0). CO, cortex; EP, epidermis. The images are representative of 8 and 9 roots from 3 independent experiments (*MEL1*::MEL1-GFP), or 9 and 6 roots from 2 independent experiments (*PIN2*::MEL1-mCherry), respectively. The images are rotated 90° counterclockwise relative to the direction of growth. (B) *PIN2*::MEL1-mCherry translational reporter localized to ectopic membrane aggregations in epidermal cells of the *pin2* mutant, instead of the apical PM as in the WT (compare to A). Introducing *PIN2::PIN2-GFP* into *PIN2::MEL1-mCherry/pin2* restored WT-like apical PM localization of MEL1-mCherry, whereas the basally localized PIN1-GFP2 expressed from the *PIN2* promoter caused MEL1-mCherry to localize basally. Images are representative of 18, 15, and 20 roots, respectively, from 4 independent experiments. Scale bars, 10 μm. See also Figure S1.

To understand the molecular mechanisms governing PIN polarity, we first investigated the inter-dependency of the remarkable colocalization of the PIN and MAB4/MEL proteins.^26^ We chose the *Arabidopsis* root meristem as the model system for (1) the feasibility of high-resolution imaging and (2) root gravitropism as a clear phenotypic readout of the function of both MAB4/MELs and PINs. To this end, we used two different complementing reporters of MEL1 (Figures S1A and S1B), which is the most broadly expressed member of the MAB4/MEL family member in the root, to examine MEL1 subcellular localization in relation to PIN2, the only PIN protein natively expressed in the root epidermis. When introduced into the *pin2* mutant background, both *MEL1::*MEL1-GFP and *PIN2::*MEL1-mCherry reporters localized to the cytoplasm (*MEL1::*MEL1-GFP) or to apolar aggregates close to the membrane (*PIN2::*MEL1-mCherry) in epidermis and lateral root cap cells, whereas in the inner cell files where other PINs are expressed, they retained the typical PIN-like polar PM localization^26^ (Figures 1A and 1B). Notably, in the presence of the wild-type (WT) *PIN2* allele or the *PIN2::PIN2-GFP* transgene, the PIN2-like apical PM localization of MEL1-GFP and MEL1-mCherry in the epidermis was restored (Figures 1A and 1B), suggesting that MEL1 might be recruited to the PM by PIN2. Previous work established that most PIN1 reporters show opposite, i.e., basal localization when ectopically expressed in the PIN2 domain.^2^ To ultimately test our hypothesis, we thus replaced the apical PIN2 with a predominantly basally localized PIN1-GFP2, which led to a pronounced basal PM localization of MEL1-mCherry in the epidermis (Figure 1B). These data indicated that MEL1 localization strictly follows the distribution of PIN1 and PIN2 within cells.

Crucially, MEL1-mCherry expressed from the *PIN2* promoter rescued gravitropic growth of the *mel1234* mutant to the same extent as native-promoter-driven MEL1-GFP and almost to WT level (Figures S1A and S1B), confirming that the subcellular localization of the *PIN2::*MEL1-mCherry reporter is physiologically relevant. Moreover, this result indicated that MAB4/MEL expression specifically in the epidermis and/or cortex cells is sufficient to rescue the reduced gravitropic growth of the *mel1234* mutant.

The finding that both PIN1 and PIN2 were capable of recruiting MEL1 to the PM in root epidermal cells led us to hypothesize that the PIN dependence of MAB4/MEL association with the PM might be a general feature of both PIN and MAB4/MEL protein families. To overcome the challenges of high-resolution PIN1 imaging in shoots and pronounced genetic redundancy with other PINs in the root,^34^ we tested this hypothesis further using transiently transformed *Arabidopsis* protoplasts. When expressed alone, PIN1-GFP localized as expected to the PM, and MAB4-RFP and MEL1-RFP localized to both the PM and cytoplasm, with MEL1-RFP showing a more punctate PM localization (Figures S1C and S1D). When co-expressed with PIN1-GFP, both MAB4-RFP and MEL1-RFP showed more pronounced localization at the PM, where they partially colocalized with PIN1-GFP (Figures S1C and S1D).

Collectively, our results show that MAB4 and MEL1 are recruited to the PM by PIN1 and PIN2 in protoplasts and *in planta*, suggesting that PIN-dependent PM recruitment of MAB4/MELs is a general functional feature of both PIN and MAB4/MEL protein families.

### PIN phosphorylation enhances MAB4/MEL recruitment to the PM

**Figure 2:**
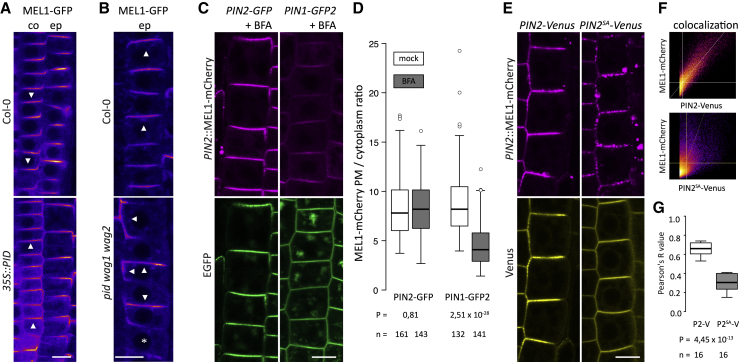
PINs recruit MEL1 to the PM in a phosphorylation-enhanced manner (A) Overexpression of PID led to a basal-to-apical switch of *MEL1*::MEL1-GFP localization in the cortex (co) cells, without affecting its apical localization in the epidermis (ep). Arrowheads indicate predominant MEL1-GFP localization in the cortex. The images are representative of 32 (Col-0) and 24 (*35S::PID*) roots analyzed in 2 independent experiments. (B) *MEL1*::MEL1-GFP in epidermal cells localized to the apical PM in Col-0 roots, whereas it displayed a range of localization defects in the *pid wag1 wag2* background, including lateral, apolar, basal, and cytoplasmic localizations. Arrowheads indicate predominant MEL1-GFP PM localization; the asterisk indicates predominantly cytoplasmic localization. The images are representative of 4 independently transformed T1 plants per genotype. (C) BFA treatment (50 μM, 1 h) had no effect on apically localized MEL1-mCherry in the epidermis in the *PIN2::PIN2-GFP* background, as reported previously for *MEL1*::MEL1-GFP in the WT.^26^ However, basally localized MEL1-mCherry in the epidermis of the *PIN2::PIN1-GFP2* background was largely dissociated from the PM upon BFA treatment (compare to Figure 1B). (D) Quantification of (C). The graph shows the ratios of PM/cytoplasm signal intensities. n indicates the number of cells from 4 different roots. The experiment was repeated independently twice with comparable results. (E) PIN2-Venus expressed from the *PIN2* promoter restored WT-like apical PM localization of MEL1-mCherry in the *pin2* mutant similar to PIN2-GFP, whereas the non-phosphorylatable PIN2^SA^-Venus largely failed to do so (compare to Figure 1B). Images are representative of 16 roots per genotype analyzed in 3 independent experiments. (F) Scatterplot representation of PIN2-Venus and MEL1-mCherry colocalization in the images shown in (E). (G) Quantification of (E) and (F). The data from all experiments were pooled; each R value represents >20 cells from one root. Scale bars, 10 μm. See also Figure S2.

Inspired by the genetic interactions between *PINs*, *MAB4/MEL*s, and *PID/WAG*s,^24^ we next examined the importance of PIN phosphorylation for MAB4/MEL recruitment to the PM using several independent strategies. First, it is well established that PID/WAGs phosphorylate the HLs of PINs and control their polarity, their overexpression leading to basal-to-apical PIN polarity shifts.^17^, ^18^, ^19^, ^20^^,^^22^^,^^35^ We also observed a basal-to-apical shift of *MEL1::*MEL1-GFP in the cortex cells of *35S::PID* roots (Figures 2A and S2G). On the other hand, in most epidermal cells of the *pid wag1 wag2* mutant, *MEL1::*MEL1-GFP displayed an intriguing range of localization defects including basal, apolar, lateral, and cytoplasmic localization, in stark contrast to the strictly apical or occasional cytoplasmic localization in Col-0 (Figures 2B and S2H). These results confirmed that the subcellular localization of MEL1 depends on the action of PID/WAGs. Furthermore, when PID-CFP was co-expressed with PIN1-GFP and MAB4- or MEL1-RFP in protoplasts, all three proteins colocalized strongly at the PM (Figures S2A and S2B). However, the PIN1^SA^-GFP fusion protein harboring S-to-A point mutations at three residues phosphorylated by AGC3 kinases (S1,2,3A)^20^^,^^22^ was inefficient at recruiting MAB4- and MEL1-RFP to the PM when compared to WT PIN1-GFP (Figures S2A and S2B). In line with PID phosphorylating PIN1 also at other residues,^20^^,^^36^ this effect of the S1,2,3A mutations was abolished when PID-CFP was co-(over)expressed (Figures S2A and S2B). These findings suggest that the recruitment of MAB4/MELs to the PM by PINs is enhanced by the action of PID and PIN phosphorylation.

Next, it has been shown that unlike PID, the PM localization of the related, basally localized AGC1 D6 protein kinase (D6PK), which also phosphorylates PINs, is highly sensitive to the ARF-GEF (guanine nucleotide exchange factor) inhibitor brefeldin A (BFA).^37^^,^^38^ As a result, BFA treatment leads to reduced phosphorylation of basally, but not apically, localized PINs.^35^ We confirmed previous observations^26^ that MEL1 localization is insensitive to BFA when colocalized apically with PIN2 in the epidermis (Figures 2C, 2D, and S2D; compare to Figures 1B and S2C). However, basally localized MEL1-mCherry in the epidermis of *PIN2::MEL-mCherry/PIN2::PIN1-GFP2/pin2*, as well as MEL1-GFP in the endodermis and stele of the native-promoter-driven *MEL1::MEL1-GFP* in the WT background, dissociated from the PM following the BFA treatment (Figures 2B, 2C, and S2D; compare to Figures 1C and S2C). Thus, the BFA sensitivities of PIN phosphorylation and MEL1 PM localization are tightly correlated. Notably, in the cortex cells, BFA treatment led to apicalization, rather than PM dissociation of basal MEL1-GFP (Figure S2D) following the rapid BFA-induced basal-to-apical transcytosis of PIN2 in these cells,^39^ confirming that MEL1 localization follows PIN localization even when PINs switch polarity (Figure 1B) also with a native-promoter-driven reporter line.

To further substantiate these findings, we made use of the fact that PM association of PID, and thus also apical PIN phosphorylation, is sensitive to the PIP-kinase inhibitor phenylarsine oxide (PAO).^40^ Accordingly, PAO treatment led to a quick and efficient depletion of apical *MEL1*::MEL1-GFP from the PM (Figures S2E and S2F). Finally, *PIN2::*PIN2^SA^-VENUS, which is not efficiently phosphorylated by PID and other AGC3 kinases due to point mutations of the three AGC3-kinase-specific phosphorylation sites (S1,2,3A),^20^^,^^22^ was incapable of restoring MEL1 localization to any of the polar PM domains of epidermal cells (Figures 2E–2G; compare to Figure 1B).

Taken together, both our protoplast and *in planta* observations show recruitment of PIN proteins to the PM by MAB4/MELs, which is strongly enhanced by PIN phosphorylation by PID and presumably other AGC kinases.

### MAB4/MELs, PINs, and PID/WAGs physically interact with each other

**Figure 3:**
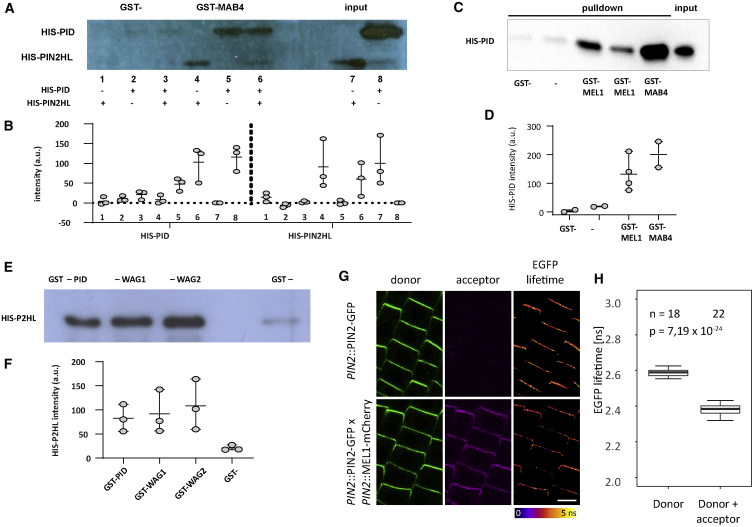
PINs, MAB4/MELs, and PID/WAGs physically interact with each other (A) *In vitro* pull-down of HIS-PIN2HL and/or HIS-PID with GST (negative control; left three lanes) or GST-MAB4 (middle three lanes). The input of HIS-tagged protein is shown in the right two lanes. The blot is representative of three independent experiments. The corresponding full western blot and Coomassie stain images are shown in Figures S3H and S3I. (B) Quantification of (A) and two independent additional experiments. Band intensities corrected for background intensity are shown. (C) *In vitro* pull-down of HIS-PID with GST-MEL1, GST-MAB4, and GST only, or non-induced GST-MEL1 lysate (−) as controls. Biologically independent lysates were used for the two GST-MEL1 lanes; the blot is representative of two technical replicates. (D) Quantification of (C) and one additional experiment. The GST-MEL1 group contains 4 data points, as two independent lysates were used in each experiment. Band intensities corrected for background intensity are shown. (E) *In vitro* pull-down of HIS-PIN2HL with GST-PID, GST-WAG1, and GST-WAG2, and GST only as control. The corresponding Coomassie stain is shown in Figure S3H. (F) Quantification of (E) and two independent additional experiments. Band intensities corrected for background intensity are shown. (G) *In vivo* FLIM-FRET imaging of *PIN2*::PIN2-GFP in the absence or presence of *PIN2*::MEL1-mCherry. Scale bar, 10 μm. (H) Quantitative analysis of (G). n indicates the total number of roots from 3 independent experiments. See also Figure S3.

To further understand the relationships between MAB/MELs, PINs, and PID/WAGs, we next asked whether members of these protein families physically interact with each other. To this end, we first performed *in vitro* pull-down assays using recombinant *E. coli*-expressed glutathione S-transferase (GST)-tagged MAB4 and HIS-tagged PIN2HL and/or PID. In these assays, HIS-PIN2HL could be pulled down with GST-MAB4, suggesting that MAB4 can indeed interact with PIN2 via its HL (Figures 3A, 3B, S3H, and S3I). Similar results were obtained when only the BTB (amino acids [aa] 22–127) or the NPH3 (aa 209–468) GST-tagged domains of MAB4 were tested for binding with HIS-PIN2HL (Figures S3A and S3B). These data show that MAB4 physically interacts with the PIN2HL, and that this interaction can be mediated with equal efficiencies by either the BTB or the NPH3 domains of MAB4, suggesting that MAB4/MELs might act as scaffolds for PIN oligomerization. Additional pull-down assays revealed physical interactions between GST-MAB4 and HIS-PID (Figures 3A–3D and S3H–S3K), GST-MEL1 and HIS-PID (Figures 3C, 3D, S3J, and S3K), and GST-PID/WAGs and HIS-PIN2HL (Figures 3E, 3F, and S3L). Furthermore, when co-incubated with HIS-PID and HIS-P2HL, GST-MAB4 pulled down both at the same time (Figures 3A, 3B, S3H, and S3I), indicating that all three proteins might co-exist in the same multiprotein complex.

To corroborate these results *in vivo*, we performed fluorescence lifetime imaging of Förster resonance energy transfer (FLIM-FRET) experiments in transiently transformed protoplasts^12^ using MEL1-GFP as donor and PIN2HL-mCherry or PID-mCherry as acceptor. These experiments confirmed that MEL1-GFP interacts with PIN2HL-mCherry as well as with PID-mCherry in plant cells (Figures S3C–S3F). Furthermore, co-expression of MAB4-RFP or MEL1-RFP with mutated PID-YFP lacking the PM-associating insertion domain^40^^,^^41^ (PID^-InsD^-YFP) led to colocalization of both proteins to intracellular tubular structures, instead of their normal PM localization (Figure S3G), further confirming the physical interaction between MAB4/MELs and PID. Furthermore, this result hints that PID can affect the localization of MAB4/MELs on its own, independent of phosphorylating PINs. Finally, we performed FLIM-FRET experiments *in planta* in root meristem epidermal cells using PIN2-GFP as donor and MEL1-mCherry as acceptor. We observed a significant reduction of fluorescence lifetime of the PIN2-GFP donor in the presence of the MEL1-mCherry acceptor (Figures 3G and 3H), confirming that MEL1 and PIN2 interact *in vivo* and *in planta*.

Taken together, our data suggest that PINs, MAB4/MELs, and PID/WAGs directly interact with each other within one multiprotein complex at the PM.

### MAB4/MELs interact with D6PK and promote PIN phosphorylation

**Figure 4:**
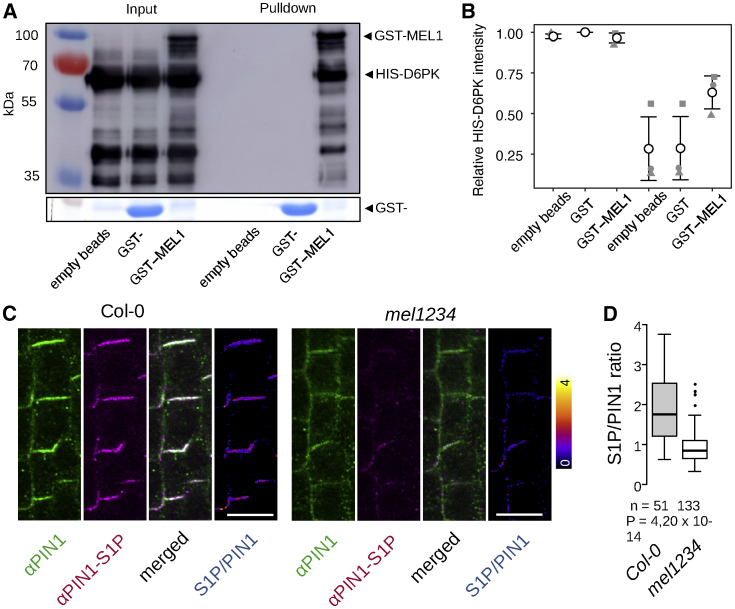
MAB4/MELs interact with D6PK and promote PIN1 phosphorylation (A) *In vitro* pull-down of HIS-D6PK with GST- (negative control; 6^th^ lane) or GST-MEL1 (last lane). The input is shown in the left three lanes. The anti-HIS western blot and the Coomassie stain showing the loading with GST- are representative of three independent experiments. Note that the loading with GST-MEL1 can be seen on the anti-HIS western blot, as the anti-HIS antibody most likely recognizes the 5HIS stretch in the MEL1 sequence (aa 72–76). (B) Quantification of (A) and two independent additional experiments. Relative HIS-D6PK band intensities are shown. (C) *In situ* immunolocalization of PIN1 (green) and PIN1 phosphorylated at the S1 residue (magenta) in wild-type (Col-0) or *mel1234* mutant root stele cells. Scale bars, 10 μm. (D) Quantitative analysis of (C). The boxplot shows the ratio of the PIN1-S1P/PIN1 signals at the PMs. n indicates the number of cells from five different roots. From 6 biological replicates in 3 independent experiments (3 with the S1P and 3 with the S4P antibody, which behaved identically under all conditions tested thus far^35^), 4 showed comparable results, 1 showed no significant difference between the genotypes, and 1 showed an opposite trend. See also Figure S4.

Our *in planta* experiments showed that MEL1 is also recruited to basally localized PIN1 (Figure 1B), which is phosphorylated primarily by D6PK from the same kinase family as PID.^35^^,^^42^ This hinted at the possibility that MAB4/MELs have similar interactions with D6PK as they do with PID. Indeed, HIS-D6PK was clearly pulled down with GST-MEL1 (Figures 4A and 4B), suggesting that MEL1 physically interacts also with D6PK.

This finding allowed us to test the relevance of MAB4/MEL-AGC kinase interaction for PIN phosphorylation *in situ* using phospho-PIN1-specific antibodies.^35^ We found that the relative proportion of phosphorylated PIN1 in its endogenous expression domain was significantly decreased in the *mel1234* mutant compared to the WT (Figures 4C and 4D). We also observed generally lower PIN1 signal levels and, in agreement with previous findings,^26^ reduced PIN1 polarity in the mutant. Nevertheless, we found no correlation between PIN1 signal intensity and the P-PIN1/PIN1 ratio in either genotype (Figures 4C and S4A), arguing against the possibility that the lower P-PIN1/PIN1 ratio in the mutant was an intensity-dependent artifact.

Because MAB4/MELs physically interact with multiple AGC kinases and the NPH3 domain is known to be phosphorylated,^31^ we next asked whether MAB4/MELs could also be direct AGC kinase targets. GST-MEL1 was not phosphorylated *in vitro* by HIS-PID, arguing against that hypothesis (Figure S4B).

Taken together, our results thus far show that (1) PINs recruit MAB4/MELs to the PM more efficiently when they are phosphorylated, and (2) MAB4/MELs promote PIN phosphorylation through interacting with the AGC kinases. These findings suggest that the PIN-MAB4/MEL-AGC kinase complex might have self-reinforcing properties thanks to positive feedback between MAB4/MEL recruitment and PIN phosphorylation.

### MAB4/MELs and PID limit PIN lateral diffusion

Having established the existence of the PIN-MAB4/MEL-AGC kinase protein complex, we next addressed the actual molecular mechanism underlying its role in mediating PIN polarity. MAB4/MELs have previously been proposed to regulate PIN internalization^26^ based on rather non-specific pharmacological manipulations of endocytosis.^43^ Nevertheless, our observations that MAB4/MEL localization followed PIN localization rather than vice versa (Figures 1A, 1B, S1C, and S1D) argued against the MAB4/MELs’ involvement in the endocytosis-dependent establishment of PIN polarity.^44^, ^45^, ^46^, ^47^ This was confirmed by our post-cytokinesis polarity establishment reporter *KNOLLE::*PIN2-GFP^47^ revealing normal apical PIN2-GFP localization in newly divided cells of the *mel1234* quadruple mutant (Figures S5). Therefore, the PIN mislocalization in the *mel1234* mutant reported previously^26^ is likely not caused by defects in PIN polarity establishment by endocytosis.

It has been proposed that the maintenance of PIN polarity depends on lateral diffusion of PINs within the PM, which is relatively slow compared to other transmembrane proteins.^15^^,^^16^^,^^48^^,^^49^ Nevertheless, experimental evidence for a causal link between lateral diffusion and PIN polarity is still lacking,^48^^,^^50^ and the mechanistic basis underlying the slower PIN lateral diffusion, besides the general requirement of the cell wall,^15^^,^^50^ also remains to be uncovered. Hence, we hypothesized that the MAB4/MEL-AGC kinase module might contribute to PIN polarity maintenance by reducing PIN lateral diffusion rate, thus limiting their escape from the respective polar domain.

Previous reports indicated that the rate of PIN2-GFP recovery in fluorescence recovery after photobleaching (FRAP) experiments in root epidermal cells did not differ between the WT and *mel1234* mutant in the presence of energy inhibitors^26^ that may directly or indirectly affect the function of MAB4/MELs and/or the AGC kinases. Nevertheless, in the time frame of minutes, FRAP dynamics of plant membrane proteins including PINs depends almost exclusively on lateral diffusion also in the absence of energy inhibitors.^48^^,^^51^ Therefore, we performed FRAP assays without any pharmacological treatments to assess the role of MAB4/MELs and AGC kinases in specific PIN lateral diffusion rates. In protoplast assays, co-expression of either PID-CFP or MAB4-RFP significantly reduced the FRAP rates of PIN1-GFP (Figures S6A and S6B), suggesting that both proteins decrease the lateral diffusion of PIN1. In line with both MAB4/MELs and PID/WAGs acting in the same molecular pathway, the effects of PID-CFP and MAB4-RFP were not additive (Figure S6A and S6B).

**Figure 5:**
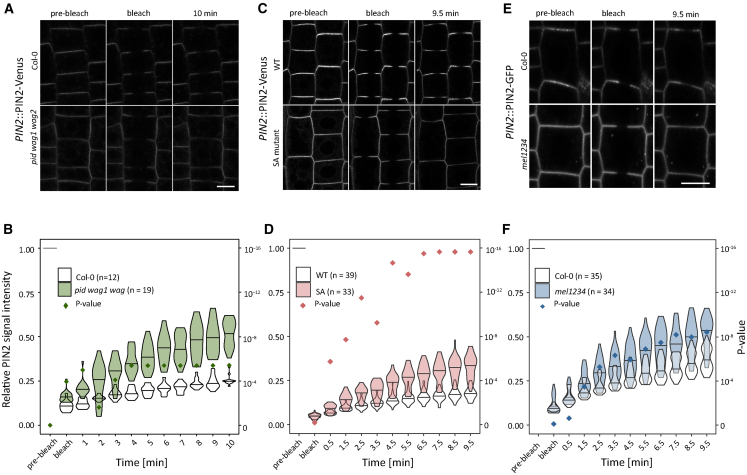
MAB4/MEL proteins and PID/WAG kinases reduce PIN lateral diffusion (A) FRAP dynamics of PIN2-Venus in Col-0 and *pid wag1 wag2* in root epidermis cells. (B) Quantitative analysis of (A). The experiment was repeated independently twice with comparable results. (C) FRAP dynamics of PIN2-Venus (WT) and PIN2^SA^-Venus (SA) in root epidermis cells. The WT images are the same as the mock control in Figure S5C. (D) Quantitative analysis of (C). The WT control is the same as the mock control in Figure S5E. The experiment was repeated independently twice with comparable results. (E) FRAP dynamics of PIN2-GFP in the WT (Col-0) and *mel1234* mutant root epidermis cells. (F) Quantitative analysis of (E). The experiment was repeated independently 3 times with comparable results. The violin plots (B, D, and F) show median values and probability density of the data after background subtraction and correction to photobleaching caused by iterative imaging. n refers to the number of cells from three different roots. Scale bars, 10 μm. See also Figures S5 and S6.

Next, we tested our hypothesis *in planta* by combining FRAP with pharmacological and genetic approaches. Treatment with PAO, which causes rapid dissociation of both PID^40^ and MEL1 (Figures S2E and S2F) from the PM, led to a significant increase in the FRAP rates of functional PIN2-Venus in its own expression domain (Figures S6C and S6E). The recovery of PIN2-Venus was significantly faster in the *pid wag1 wag2* triple mutant as compared to Col-0 (Figures 5A and 5B). Furthermore, the point mutations in PIN2^SA^-Venus, which render it largely non-phosphorylatable by the PID/WAGs,^22^ also caused an increase in FRAP rates compared to the WT PIN2-Venus control (Figures 5C and 5D). Finally, we observed a significantly higher PIN2-GFP FRAP rate in the *mel1234* mutant as compared to the Col-0 control (Figures 5E and 5F). Because this finding directly contradicted previously reported results,^26^ we aimed to confirm it with a complementary *in planta* gain-of-function experiment, and indeed observed that estradiol-inducible overexpression of MEL1-TagRFP decreased the recovery of PIN2-GFP (Figures S6D and S6F).

Taken together, our FRAP assays, involving both loss- and gain-of-function approaches, two model systems, and different members of the respective protein families, are all consistent with the hypothesis that the PID-MAB4/MEL module consolidates PIN polarity by limiting PIN lateral diffusion-based escape from the respective polar domain.

### Diffusion rates of MEL1 are fast compared to PIN

**Figure 6:**
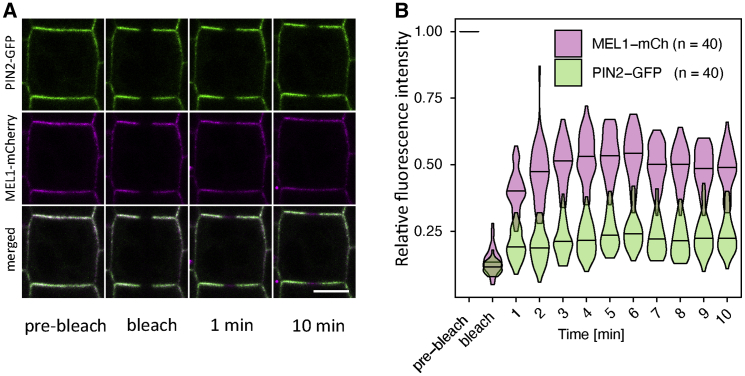
MEL1-mCherry diffuses fast compared to PIN2-GFP (A) FRAP dynamics of PIN2-GFP and MEL1-mCherry in the same root epidermis cells. Scale bar, 10 μm. (B) Quantitative analysis of (A). n indicates the number of cells from 3 different roots. The experiment was repeated independently twice with comparable results. The violin plots show median values and probability density of the data after background subtraction and correction to photobleaching caused by iterative imaging. n refers to the number of cells from three different roots.

Collectively, our data suggest that PINs, MAB4/MELs, and AGC kinases form a single self-reinforcing multiprotein complex at the PM, which limits the lateral diffusion-based escape of PINs from their respective polar domains. This is reminiscent of the positive feedback loop in the Cdc42-dependent symmetry-breaking pathway in yeast, where the active GTP-Cdc42 recruits the BEM1 protein complex, which includes its own GEF activator.^13^ A key feature of this system that enables it to generate polarity is the difference between the slow and fast diffusion rates of the membrane-bound GTP-Cdc42 and the cytoplasmic components of the Bem1 complex, respectively.^13^^,^^14^ This comparison then implies that if the PIN-MAB4/MEL-AGC kinase polarity module described here operates in a similar manner, the soluble MAB4/MELs would have to diffuse fast in comparison to the membrane-bound PINs. We have tested this prediction by a dual-color FRAP experiment in the *PIN2::PIN2-GFP x PIN2::MEL1-mCherry* line, and indeed observed that the recovery of the MEL1-mCherry signal was very fast in comparison to the relatively slow recovery of PIN2-GFP (Figures 6A and 6B).

## Discussion

### Self-reinforcing PIN-MAB4/MEL-AGC kinase module for PIN polarity maintenance

Plants exhibit amazing developmental plasticity, which relies on the plant-specific patterning mechanism of polarized flow of the plant hormone auxin through tissues. The key components of this mechanism are PIN auxin transporters that determine the directionality of auxin transport through their polar subcellular localization.^2^ In certain situations, such as tropic responses or wound healing, the PIN polar distribution at the PM needs to quickly change in response to endogenous and environmental cues, thus redirecting auxin fluxes.^8^, ^9^, ^10^, ^11^, ^12^ However, for the maintenance of some stem cell niches, PIN polarity has to remain stable.^6^^,^^7^^,^^52^ Thus, one of the key enigmas of plant cell biology is how to maintain stable PIN polar localization patterns while allowing them to flexibly change when needed.^3^

Phosphorylation by the PID/WAG kinases is tightly linked to PIN apical-basal polarity^17^, ^18^, ^19^, ^20^^,^^22^ as well as to dynamic polarity changes in response to light or gravity.^8^, ^9^, ^10^ Nevertheless, the mechanism by which phosphorylation of PINs regulates their polarity remained unclear and controversial.^32^^,^^33^^,^^35^ MAB4/MEL proteins were identified as additional PIN polarity regulators and, apart from evidence that they act in the same genetic pathway as PID/WAGs, their molecular function remained entirely unclear.^24^, ^25^, ^26^, ^27^

**Figure 7:**
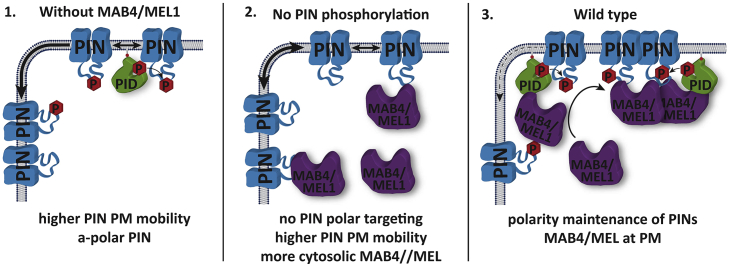
Proposed model of PID-MAB4/MEL positive feedback loop mediating PIN polarity maintenance through limiting lateral diffusion Left: PID can interact with and phosphorylate the PIN hydrophilic loop (PINHL; P indicates a phospho-residue). However, without MAB4/MELs, there is still increased lateral diffusion of PINs. Middle: without PID, unphosphorylated PINHLs do attract MAB4/MELs but at a much lower efficiency, leading to more lateral diffusion. Right: when all three are present, the interaction of PID with the PINHL and the subsequent phosphorylation attract MAB4/MELs that act as scaffolds to form PIN/PID/MAB4 complexes, increasing PINHL phosphorylation and limiting lateral diffusion by PIN complex formation.

Here we report that PID/WAG and MAB4/MEL proteins are part of the same, plant-specific mechanism for PIN polarity maintenance. We show that initial PIN polar targeting does not depend on MAB4/MEL localization, as we had hypothesized based on our previous finding that MEL1-GFP recruitment to the new PM after cytokinesis precedes the re-establishment of PIN2 polarity.^47^ Instead, we found that PINs recruit the MAB4/MELs to the PM by protein-protein interactions. The efficiency of MAB4/MEL recruitment is tightly correlated with the phosphorylation of PINs by PID, WAG, and D6PK AGC kinases. MAB4/MELs limit the lateral diffusion-based escape of PINs from their polar domain, and at the same time interact with the AGC kinases themselves and promote PIN phosphorylation. The PIN-MAB4/MEL-AGC kinase complex thus appears to have self-reinforcing properties, which would provide a molecular mechanism enabling plants to maintain a stable polar subcellular PIN localization pattern, which can still be quickly adjusted in response to environmental or developmental cues (Figure 7).

This model assumes and predicts the existence of additional molecular players to constrain the activity of the AGC-MAB4/MEL module to the apical/basal PM domains and prevent it from stabilizing PINs at the lateral ones. Such factors might include polarized PIN secretion,^48^ spatial regulation of the activity of the apolar PID/WAGs,^33^ or additional requirements for MAB4/MEL recruitment, such as specific lipid composition of different PM domains or other hypothetical cell polarity factors. In newly divided cells, the re-establishment of apical polarity of PIN2 strictly depends on PID/WAGs but not MAB4/MELs (Figure S5), and MEL1 does not localize to the cell plate.^47^ It is therefore conceivable that a sufficient proportion of the PIN2 pool needs to be phosphorylated to allow the recruitment of MEL1, which then helps to reinforce and maintain the established PIN2 polar distribution pattern. Uncovering the nature and order of events in the initial establishment of PIN polarity, and the role of the apolar PID/WAG kinases therein, remains an exciting challenge for future investigations.

Interestingly, the PIN2HL interacts with both the BTB and NPH3 domains of MAB4 (Figures S3A and S3B). A role of the BTB domain in mediating protein-protein interactions is well established (Robert et al.^29^ and references therein), but the function of the plant-specific NPH3 domain has until now remained unclear.^31^ Our results indicate that the NPH3 domain also mediates protein-protein interactions, and that therefore MAB4/MELs might act as scaffolds for multimerization of PIN proteins^53^ while at the same time interacting with PIN-phosphorylating kinases. Such a positive feedback loop would create a self-reinforcing PIN-MAB4/MEL-AGC kinase complex at the PM, which limits the lateral diffusion-based escape of PINs from their polar domains, thus contributing to the maintenance of stable PIN polar distribution patterns (Figure 7). Concomitantly, thanks to its ability to amplify small initial differences in PIN abundance and/or phosphorylation levels, the PIN-MAB4/MEL-AGC kinase module would also allow for dynamic changes of PIN polar localization, which occur during tropic responses and were shown to depend on PIN phosphorylation by the PID/WAG kinases.^8^, ^9^, ^10^

How precisely the PIN-MAB4/MEL-AGC kinase complex limits PIN lateral diffusion remains to be discovered. One plausible scenario is that the putative scaffolding function of MAB4/MELs would induce PIN oligomerization, and the resulting PIN oligomers would be less mobile simply due to their size,^16^^,^^49^^,^^54^ similar to the oligomerization-dependent mechanism of polarity establishment of the SOSEKI proteins.^55^^,^^56^ Super-resolution analysis of PIN distribution in different *MAB4/MEL* and *PID* loss- and gain-of-function backgrounds should be carried out to test this hypothesis.

### Implications and future directions

Our results provide a clear mechanistic explanation for the convergent agravitropic *pin2*-like root phenotypes of the *pidwag1wag2* and *mel1234* loss-of-function mutants and of the *pin2* mutant expressing the non-phosphorylatable PIN2^SA^-Venus variant from the *PIN2* promoter.^5^^,^^26^^,^^22^ All these mutant combinations prevent or reduce the formation of the PIN2-MAB4/MEL-PID/WAG complex, thus hampering PIN2 polarity through increasing the lateral diffusion-based escape of PIN2 from the apical PM domain. Different *pid* and *mab4/mel* mutant combinations phenocopy *pin* mutants also in other developmental contexts.^23^^,^^25^^,^^28^ In our work, any of the PIN, MAB4/MEL, and PID/WAG homologs were largely interchangeable in various experimental setups. This suggests that our model can probably be extrapolated to the general mode of action of all three protein families throughout development. Nevertheless, this hypothesis should be further validated by detailed *in planta* analysis of the roles of PID/WAGs and MAB4/MELs in other PIN-regulated developmental processes. In the inflorescence meristem, MAB4/MELs are needed for inward repolarization of PIN1 in the L1 surface layer, whereas PID is required already for the initial polarization of PIN1 toward the center of each primordium,^27^ highlighting an additional, MAB4/MEL-independent role of PID/WAGs already during the initial PIN polarity establishment.^47^ Furthermore, given the indicated role of phosphorylation in PIN activation,^33^^,^^36^^,^^57^ it would also be interesting to address the role of the PIN-MAB4/MEL-AGC kinase module in PIN transport activity in future research.

Why PIN phosphorylation by PID/WAGs promotes their apical localization,^17^, ^18^, ^19^, ^20^^,^^22^ whereas the related D6PK and its D6PK-like (D6PKL) homologs phosphorylate PINs at partially overlapping residues as PID without imposing changes in PIN polarity,^20^^,^^35^^,^^36^^,^^58^ is currently not understood and a matter of debate.^32^^,^^33^ Our findings here imply that AGC-kinase-mediated PIN phosphorylation acts in concert with MAB4/MELs to reinforce and maintain PIN localization at the PM domain where phosphorylation occurs. This model can thus reconcile the above-mentioned discrepancy through the differential subcellular localization of the kinases, because PID/WAGs are apolar^22^^,^^37^ and might act preferentially at the apical PM through unknown regulatory mechanisms,^33^^,^^47^ whereas D6PK shows strictly basal localization.^38^^,^^58^ These localization patterns imply that the overexpression of PID, but not of D6PK, can lead to PIN phosphorylation events at the apical PM, and thereby to the stabilization and, in the long-term, promotion of apical PIN localization in concert with MAB4/MELs. The observations that prolonged treatment with the ARF-GEF GNOM inhibitor BFA leads to basal-to-apical PIN polarity shifts^39^ are also consistent with this model, as BFA causes rapid PM dissociation of basal D6PK but not of apolar PID,^35^^,^^58^ and therefore increases the relative incidence of PIN phosphorylation at the apical PM. The differential, although overlapping, phosphosite preference of the different classes of AGC kinases could also contribute to their contrasting effects on PIN apical localization, which depends on concurrent phosphorylation of all three S1, S2, and S3 residues.^20^

### Positive feedback loops and lateral diffusion as recurrent topics in cell polarity

In animal systems, protein polarity maintenance often depends on diffusion barriers that prevent cargoes from escaping their respective polar domains.^59^^,^^60^ In plants, however, only a few highly specialized cell types possess similar structures,^61^^,^^62^ and hence the general mechanism of polarity maintenance in plant cells remained conceptually unclear.^63^ The PIN-MAB4/MEL-AGC kinase polarity complex described here locally modifies PIN lateral diffusion rates, thereby providing a plant-specific, diffusion-barrier-independent mechanism of protein polarity maintenance. It is conceivable that the plant protophloem-specific BRX-PAX-PIP5K module,^64^ as well as other plant polar cargoes,^65^^,^^66^ are regulated by a similar phosphorylation-dependent regulation of lateral diffusion, and it will be exciting to unravel the unknown features of the underlying molecular mechanisms in the future.

The PIN-MAB4/MEL-AGC module is mechanistically similar to the Cdc42-dependent symmetry-breaking pathway in yeast^13^^,^^14^^,^^67^ but utilizes different molecular components. Polar localization of the recently discovered SOSEKI proteins also seems to depend on their slow lateral diffusion achieved through yet another molecular mechanism.^56^ Therefore, positive feedback in protein complex assembly and limited lateral diffusion appear as key mechanisms in generating cellular polarity across kingdoms and polarity systems.

## STAR★Methods

### Key resources table

**Table:** 

REAGENT or RESOURCE	SOURCE	IDENTIFIER
**Antibodies**		

anti-HIS-HRP	Roche	Cat# 11965085001; RRID: AB_514487
Rabbit αPIN1	^68^	N/A
Rabbit αPIN2	^69^	N/A
Rabbit αPIN4	^70^	N/A
rabbit αPIN1-S1P	^35^	N/A
rabbit αPIN1-S4P	^35^	N/A
guinea pig αPIN1	^35^	N/A
mouse αGFP	Sigma	Cat# G6539; RRID: AB_259941
Alexa Fluor 488-conjugated goat αMouse IgG	Invitrogen	Cat# A28175; RRID: AB_2536161
Alexa Fluor 647-conjugated goat αGuinea pig IgG	Invitrogen	Cat# A-21450; RRID: AB_141882
Cy3-conjugated sheep αRabbit IgG	Sigma	Cat# AP510C

**Bacterial and virus strains**		

*Escherichia coli* DH5a	Lab stock	N/A
*E.coli* BL21 (DE3)	New England Biolabs	Cat# C2527H
*Agrobacterium tumefaciens* GV3101	Lab stock	N/A

**Chemicals, peptides, and recombinant proteins**		

Brefeldin A (BFA)	Sigma	Cat# B7651
Phenylarsine oxide (PAO)	Sigma	Cat# P3075
GST-MEL1	This study	N/A
His-PID	^20^	N/A
GST-MAB4	This study	N/A
His-PIN2HL	^42^	N/A
GST-	N/A	N/A
GST-PID	^22^	N/A
GST-WAG1	^22^	N/A
GST-WAG2	^22^	N/A
His-D6PK	^42^	N/A
GST-^MAB4^BTB	This study	N/A
GST-^MAB4^NPH3	This study	N/A

**Critical commercial assays**		

γ -[32P]-ATP	PerkinElmer	Cat# NEG502A001MC

**Experimental models: organisms/strains**		

*Arabidopsis thaliana* Col-0	N/A	N/A
*A. thaliana eir1-1*	^5^	N/A
*A. thaliana PIN2::PIN2-GFP*	^71^	N/A
*A. thaliana PIN2::PIN2-GFP/eir1-4*	^69^	N/A
*A. thaliana PIN2::PIN1-GFP2*	^2^	N/A
*A. thaliana PIN2::PIN2-Venus/eir1-1*	^22^	N/A
*A. thaliana PIN2::PIN2*^*SA*^*-Venus/eir1-1*	^22^	N/A
*A. thaliana pid wag1 wag2*	^22^	N/A
*A. thaliana KNOLLE::PIN2-GFP*	^47^	N/A
*A. thaliana MEL1::MEL1-GFP*	^47^	N/A
*A. thaliana mel1234*	^26^	N/A
*A. thaliana PIN2::PIN2-GFP/mel1234*	^26^	N/A
*A. thaliana 35S::PID*	^19^	N/A
*A. thaliana KNOLLE::PIN2-GFP/mel1234*	This study	N/A
*A. thaliana MEL1::MEL1-GFP/mel1234*	This study	N/A
*A. thaliana MEL1::MEL1-GFP/eir1-1*	This study	N/A
*A. thaliana PIN2::MEL1-mCherry*	This study	N/A
*A. thaliana PIN2::MEL1-mCherry/eir1-1*	This study	N/A
*A. thaliana PIN2::MEL1-mCherry/mel1234*	This study	N/A
*A. thaliana MEL1::MEL1-GFP/pid wag1 wag2*	This study	N/A
*A. thaliana PIN2::PIN2-Venus/pid wag1 wag2*	This study	N/A
*A. thaliana PIN2::MEL1-mCherry/PIN2::PIN2-GFP/eir1-1/4*	This study	N/A
*A. thaliana PIN2::MEL1-mCherry/PIN2::PIN1-GF2/eir1-1*	This study	N/A
*A. thaliana PIN2::MEL1-mCherry/PIN2::PIN2-Venus/eir1-1*	This study	N/A
*A. thaliana PIN2::MEL1-mCherry/PIN2::PIN2*^*SA*^*-Venus/eir1-1*	This study	N/A
*A. thaliana MEL1::MEL1-GFP/35S::PID*	This study	N/A
*A. thaliana XVE≫MEL1-TagRFP/PIN2::PIN2-GFP*	This study	N/A

**Oligonucleotides**		

For primers used in this study, see Table S1	This study	N/A

**Recombinant DNA**		

Plasmid *35S::PIN1-GFP*	This study	N/A
Plasmid *35S::MAB4-mRFP*	This study	N/A
Plasmid *35S::MEL1-mRFP*	This study	N/A
Plasmid *35S::PID-CFP*	This study	N/A
Plasmid *35S::MEL1-GFP*	This study	N/A
Plasmid *35S::PIN2HL-mCherry*	This study	N/A
Plasmid *35S::PID-mCherry*	This study	N/A
Plasmid *35S::PID-YFP*	This study	N/A
Plasmid *35S::PID(-insDom)-YFP*	This study	N/A

**Software and algorithms**		

FIJI	^72^	https://fiji.sc/
ICY bioimage analysis	^73^	https://icy.bioimageanalysis.org/
R	^74^	https://www.R-project.org/
R-studio	^75^	http://www.rstudio.com
ggplot2 package for R	^76^	http://ggplot2.org
LifetimeAnalyser	This study	https://seafile.ist.ac.at/d/5c6033ab9fa9412c9a27/

### Resource Availability

#### Lead contact

Further information and requests for resources and reagents should be directed to and will be fulfilled by the Lead Contact, Jiří Friml (jiri.friml@ist.ac.at).

#### Materials availability

DNA constructs and transgenic *Arabidopsis* seeds generated in this study are available from the Lead Contact, Jiří Friml, upon request.

#### Data and code availability

The “LifetimeAnalyser” script for the analysis of FLIM-FRET data generated in this study is publicly available at the IST Austria data repository (https://seafile.ist.ac.at/d/5c6033ab9fa9412c9a27/). The raw data and code used for other analyses are available from the Lead Contact, Jiří Friml, upon request.

### Experimental Model and Subject Details

#### Plant material and growth conditions

Seeds were surface-sterilized by chlorine vapor, sown on 1/2 Murashige-Skoog medium supplemented with 1% sucrose and 1% agar and grown *in vitro* under long day conditions. The transgenic and mutant lines *eir1-1*,^5^ *PIN2::PIN2-GFP*,^71^ *PIN2::PIN2-GFP/eir1-4*,^69^ *PIN2::PIN1-GFP2*,^2^ *PIN2::PIN2-Venus, PIN2::PIN2*^*SA*^*-Venus, pid wag1 wag2*,^22^ *KNOLLE::PIN2-GFP*, *MEL1::MEL1-GFP*,^47^ *mel1234, PIN2::PIN2-GFP/mel1234*^26^ and *35S::PID*^19^ have been described previously. The lines *KNOLLE::PIN2-GFP/mel1234*, *MEL1::MEL1-GFP/mel1234*, *MEL1::MEL1-GFP/eir1-1, PIN2::MEL1-mCherry*, *PIN2::MEL1-mCherry/eir1-1, PIN2::MEL1-mCherry/mel1234, MEL1::MEL1-GFP/pid wag1 wag2, PIN2::PIN2-Venus/pid wag1 wag2 and XVE≫MEL1-TagRFP/PIN2::PIN2-GFP* were obtained by transforming the constructs into the respective background by the floral dip method.^77^ *MEL1::MEL1-GFP/eir1-1* and *PIN2::MEL1-mCherry/eir1-1* were crossed with Col-0 and the respective *PIN1/PIN2-XFP/pin2* lines described above and F1 seeds heterozygous for each of the two fluorescent reporters and homozygous for the *eir1-1* mutation (or *eir1-1 eir1-4* biallelic) were used. *MEL1::MEL1-GFP* was crossed with *35S::PID* and F2 seedlings were used for analysis. For the *in planta* FLIM-FRET experiments, homozygous *PIN2::PIN2-GFP* and segregating T2 *PIN2::MEL1-mCherry* lines were used, resulting in a 1:1 ratio of plants expressing only the donor and both the donor and acceptor fluorescent markers. Phenotype analysis was performed as described previously.^47^

### Method Details

#### Molecular cloning

Cloning of plant expression constructs was performed using the Gateway technology (Invitrogen). *PIN2::MEL1-mCherry* was obtained by recombination of the entry clones *pPIN2(pDonrP4-P1r)*,^78^ *MEL1(pDonr221)*^47^ and *mCherry(pDonrP2r-P3)*^46^ into the destination vector *pH7m34GW,0*. *MEL1(pDonr221)* was recombined with *PK7FWG2* to yield *35S::MEL1-GFP*. To generate *35S::PIN2HL-mCherry* and *35S::PID-mCherry*, the PIN2 central hydrophilic loop sequence (corresponding to amino acid residues 157 - 484) and the PID coding sequence, respectively, were cloned into *pDonr221* and subsequently recombined into *p2GWCh7,0*.^79^ To generate *XVE≫MEL1-TagRFP,* the *MEL1* genomic fragment was subcloned into *pENTR/D-TOPO* (Thermo Fisher Scientific). A PCR-amplified *TagRFP* coding sequence was subsequently inserted in frame to the 3′ end of MEL1 coding sequence by the In-Fusion cloning reaction (TaKaRa) to generate *MEL1-TagRFP(pENTR/D-TOPO),* which was recombined into *pMDC7*.^80^

For expression in protoplasts, *MAB4/MEL* genes were amplified from cDNA-based clones provided by the Riken Institute in Japan, using primers with attB sites. PCR products were cloned into pDONR207 by BP reaction and subsequently into pART7^81^ modified with a Gateway cloning cassette between a CaMV *35S* promoter and a *RFP* coding region with a Gateway LR reaction. The cloning procedure for *PID* was described previously^22^ and the pDONR207:PID vector was recombined into pART7:35S:CFP.

The pBluescript-based *35S::PIN1-GFP* vector was used for protoplast transformation^45^ and the PIN1HL and PIN2HL and S-to-A mutant constructs^20^ were described before. *pDONR:PID*^22^ was used to recombine into *pART7:35S:CFP/YFP/RFP* vectors. *PID*^*+InsDom*^ and *PID*^*-InsDom*^ were created by introducing *Sgs*I and *Bsp*TI restriction sites at the N- and C-terminal border respectively of the PID insertion domain by site-directed mutagenesis (performed as described previously^82^ with minor modifications) of the pDONR207:PID Gateway entry vector and subsequently deleting the insertion domain by restriction and ligation.

To generate the *GST-MEL1* and *GST-MAB4*, the coding sequences were amplified and cloned into pGEX-4T-1 and pGEX-GST (both GE Healthcare), respectively. For *His-PID* constructs, the *PID* coding sequence was cloned into the pET28a (GE Healthcare) or pET16H-HIS (Novagen) vectors. The BTB and NPH3 domains of MAB4 were amplified from cDNA using attB primers and Gateway recombination into pDONR207 and recombined into pGEX-GST to yield GST-BTB and GST-NPH3. The construction of the GST-PID/WAG1/WAG2 and HIS-PIN2HL^22^ and HIS-D6PK^42^ constructs have been described previously.

The sequences of all primers used can be found in Table S1.

#### Protoplast isolation and transformation

*Arabidopsis thaliana* Col-0 cell suspension cultures were used for protoplast preparations. In experiments presented in Figures 1, S5, and S6, protoplasts were prepared as described previously^83^ with minor modifications. Briefly: four-to-six day old cultures were diluted 5-fold in Cell Medium (30 g /L sucrose, 3.2 g/L Gamborg’s B5 basal medium with mineral organics, adjusted to pH 5.8 with KOH and sterilized by autoclaving), incubated overnight and used for protoplast isolation. Cellulase and macerozyme digestion of cell walls was performed during at least three hours at 27°C degrees in darkness with very gently agitation. Protoplasts were isolated using a sterile 63 μm steel sieve. Following PEG transfection with 10μg plasmid DNA per 1^∗^10^6^ protoplasts, the cells were incubated at 25°C in the dark for 16-18 hours before observation or additional treatments.

In experiments presented in Figure S3, protoplasts were prepared as described previously^84^^,^^85^ with minor modifications. Briefly: 3-day-old *Arabidopsis* root suspension cell cultures were pelleted, resuspended in GM buffer (Murashige-Skoog Basal Salt Mixture 4.4 g/l, 0.17M glucose, 0.17M mannitol, pH5.5) with 1% cellulase and 0.2% macerozyme and incubated for 4h in darkness with gentle shaking. Protoplasts were separated by sucrose gradient centrifugation, concentration-adjusted to 10^8^ cells/ml, and incubated for 1h in darkness with 12-15 μg of plasmid DNA in the presence of PEG, followed by a wash and overnight incubation in GM buffer.

#### Pull-down and western blot

For the production of protein-containing *E. coli* lysate, expression vectors were transformed into strain BL21 (DE3) and selected for strong induction of recombinant protein. 5ml of overnight culture was added to 50ml of LC medium supplemented with antibiotics and grown to an OD-600 of 0.4-0.8. The cultures were subsequently induced for 4 hours with 0.4 mM IPTG. After induction, the cultures were centrifuged at 4000 rpm for 20 min and the pellets were stored at −20°C. Pellets were resuspended in fresh extraction buffer (1x PBS, 2 mM EDTA, 2 mM EGTA, 2 mM DTT, 150 mM NaCl pH 8, supplemented with 1 mM PMSF with protease inhibitor tablets from Pierce and 1 mg/ml lysozyme) and incubated for 1 hour at 4°C on a rocking table. Cells were sonicated (on ice) and cell debris was centrifuged at 14000rpm at 4°C for 30 minutes. After addition of 1% triton the supernatant (containing the expressed protein) was divided into 100μl aliquots. For pull-down, 100μl of GST-tagged lysate (in total 500 μl extraction buffer) was bound to glutathione agarose beads for 2 hours at RT. 100 μl of each HIS-tagged protein was loaded onto the beads and binding buffer (50 mM Tris-HCl pH 6, 200 mM NaCl, 0.1% Tween 20) was added to a total reaction volume of 400 μl. The samples were then incubated at RT for 2 hours on an Eppendorf rotator. After incubation, the resin was washed 3 times in 500 μl wash buffer (25 mM Tris-HCl pH 8, 10% glycerol, 300 mM NaCl, 20 mM imidazole, 0.05% Tween 20) with in-between centrifugation at 4000 rpm for 3 minutes. 40 μl of 1x SDS-PAGE loading buffer (40% glycerol, 240 mM Tris-HCl pH 6.8, 8% SDS, 0,04% bromophenol blue, 5% β-mercaptoethanol) was added to the precipitated resin and samples were boiled at 99°C for 10 minutes. The boiled resin was spun down and the supernatant was loaded to a 12,5% / 4,5% manually cast or 15% / 4% TGX pre-cast (Biorad, Figure 3C) polyacrylamide gels for SDS-PAGE. Blotting was performed in a transblot semi-dry setup using top (60 mM Tris, 40 mM CAPS, pH 9.6 + 0.1% SDS) and bottom buffer (60 mM Tris, 40 mM CAPS, pH 9.6 + 15% MeOH) onto a PVDF membrane. For the blot in Figure 3C, a biorad transblot turbo semi-dry blotting device with supplied buffers and PVDF membrane was used. Blocking was performed with 5% Elk-brand milk powder in TBS at 4°C overnight. The membrane was then probed for an hour at RT with an anti-HIS-HRP antibody (Roche,1:1000 diluted) and chemiluminescence (LumiGlo, Cell Signaling) was detected using and X-Ray film (Fuji) or the GelDoc imager (Biorad, Figure 3C).

#### *In vitro* protein kinase assay with [*γ*-32P] ATP

To express recombinant GST-MEL1 and His-PID proteins, the respective constructs were transformed into *E. coli* BL21 (DE3) cells for protein expression. Cultures at OD600 of 0.6 were induced with 0.5mM IPTG at 16°C overnight. Proteins were purified using Glutathione agarose for GST-MEL1 and Ni-NTA His binding resin for His-PID following the manufacturer’s instructions (Thermo Scientific). Purified proteins were analyzed by SDS-PAGE and visualized by Coomassie brilliant blue staining (Bio-Rad). *In vitro* protein kinase assay with [γ-^32^P] ATP was carried out as previously reported with minor modifications.^42^ Recombinant GST-MEL1 (2 μg) and His-PID (5 μg) proteins were incubated together in 25 μL kinase reaction buffer [50 mM Tris-HCl pH 7.5, 10 mM MgCl_2_, 1 mM DTT, 0.1 mM ATP, 10 μCi [γ-^32^P] ATP (NEG502A001MC; Perkin-Elmer)] at 25°C for 1 h. Afterward, the reactions were terminated by adding SDS loading dye, and samples were resolved by 10% SDS-PAGE. The phosphorylated bands indicated by ^32^P signal was visualized by autoradiography with a phosphor-plate on a Fujifilm FLA 3000 plus DAGE system.

#### Imaging and image analysis

Imaging of protoplasts (Figures 1, S5, and S6) was performed as follows: 150-200 μL protoplasts in protoplast medium were pipetted into an 8-well chambered coverslip (Lab-Tek). Images were taken with a Zeiss LSM5 AxioImager inverted microscope. FRAP was performed by bleaching until the intensity reached < 5% of original intensity using the ZEN-software built in bleach function (https://www.zeiss.de/zen).

All other confocal imaging was performed using Zeiss LSM700, LSM800 or LSM880 inverted microscopes. For live imaging, 4-day-old, chambered coverslip (Lab-Tek)-mounted seedlings were used. Immunofluorescence staining was performed as described previously;^86^ following antibodies were used at the dilutions indicated: rabbit αPIN1,^68^ 1:1000; rabbit αPIN2,^69^ 1:1000; rabbit αPIN4^70^ 1:250; rabbit αPIN1-S1P, 1:100; rabbit αPIN1-S4P, 1:400; guinea pig αPIN1,^35^ 1:1000; mouse αGFP (Sigma), 1:1000; Alexa Fluor 488-conjugated goat αMouse IgG (Thermo Fisher), 1:600; Cy5-conjugated goat αRabbit IgG (Thermo Fisher), 1:600; Alexa Fluor 647-conjugated goat αGuinea pig IgG (Invitrogen), 1:600; Cy3-conjugated sheep αRabbit IgG (Sigma Aldrich), 1:600. BFA and PAO (both Sigma) treatments were applied by transferring the seedlings onto a small slice of agar medium containing the respective chemical as described previously;^47^ the DMSO stock solution and final concentrations were 50mM/50 μM (BFA) and 60mM/30 or 60 μM (PAO).

*In planta* FRAP experiments and assessment of PIN2 polarity re-establishment from time-lapse imaging of *KNOLLE::PIN2-GFP*-expressing plants were performed as described previously.^47^

FLIM-FRET experiments were performed using a TriM Scope II inverted 2-photon microscope equipped with a FLIM X16 TCSPC detector for time correlated single photon counting (LaVision BioTec).

### Quantification and Statistical Analysis

Image analysis was performed with the FIJI distribution of ImageJ,^72^ and the ICY bioimage analysis software based on ImageJ for Figures S2C, S2D, S3A, S3B, S6A, and S6B.^73^ Basic tools were used for signal intensity measurements in confocal images and root measurements (Figure S1B).

For colocalization analysis (Figures 2F and 2G), PMs were segmented by a threshold mask (keeping the threshold value constant within an experiment); colocalization scatterplots were generated with the *Colocalization threshold* and Pearson’s R-values with the *Coloc2* plugins of FIJI.

FLIM-FRET data was analyzed as follows: Fluorescence lifetime image stacks (150 slices, with 0,082 ns time interval) were acquired, and a threshold mask was created from the sum projection of each stack in FIJI^72^ to segment the apical PM domains. All pixels within the masked area were then pooled and averaged at each time point of the FLIM stack. The intensity at t = 0 was normalized and a simple exponential decay [I(t) = A^∗^exp(-t/lambda)+offset] was fitted to the data. The “LifetimeAnalyzer” MATLAB-based script that generates a single lifetime value for each image based on the source FLIM stack and the threshold mask can be found at https://seafile.ist.ac.at/d/5c6033ab9fa9412c9a27/.

Data was handled with Microsoft Excel. Statistical analysis and plotting was performed with R version 3.6.2, using RStudio version 1.2.5033 and the ggplot2 package.^74^, ^75^, ^76^ Normal distribution of data was assessed with the Shapiro test. If not mentioned otherwise, P values were calculated with Student’s t test (with equal/unequal variance settings following the result of the F-test) or with the Wilcoxon test for data with and without normal distribution, respectively. Box-plots represent median, 1st and 3rd quartile; the whiskers extend to data points < 1,5 interquartile range away from the 1st/3rd quartile, outliers are shown as empty circles. Figures were assembled in LibreOffice Draw.

## Supplemental information

Document S1. Figures S1–S6 and Table S1

Document S2. Article plus supplemental information
