# Supplementary material for: AGC kinases and MAB4/MEL proteins maintain PIN polarity by limiting lateral diffusion in plant cells
Source: Curr Biol. 2021 May 10;31(9):1918–1930.e5. doi: 10.1016/j.cub.2021.02.028 (PMC8112251; doi:10.1016/j.cub.2021.02.028)
Supplement: Document S1. Figures S1–S6 and Table S1 [file mmc1.pdf]

Current Biology, Volume *31*

Supplemental Information

AGC kinases and MAB4/MEL proteins maintain PIN polarity by limiting lateral diffusion in plant cells

Matouš Glanc, Kasper Van Gelderen, Lukas Hoermayer, Shutang Tan, Satoshi Naramoto, Xixi Zhang, David Domjan, Ludmila Včelařová, Robert Hauschild, Alexander Johnson, Edward de Koning, Maritza van Dop, Eike Rademacher, Stef Janson, Xiaoyu Wei, Gergely Molnár, Matyáš Fendrych, Bert De Rybel, Remko Offringa, and Jiří Friml

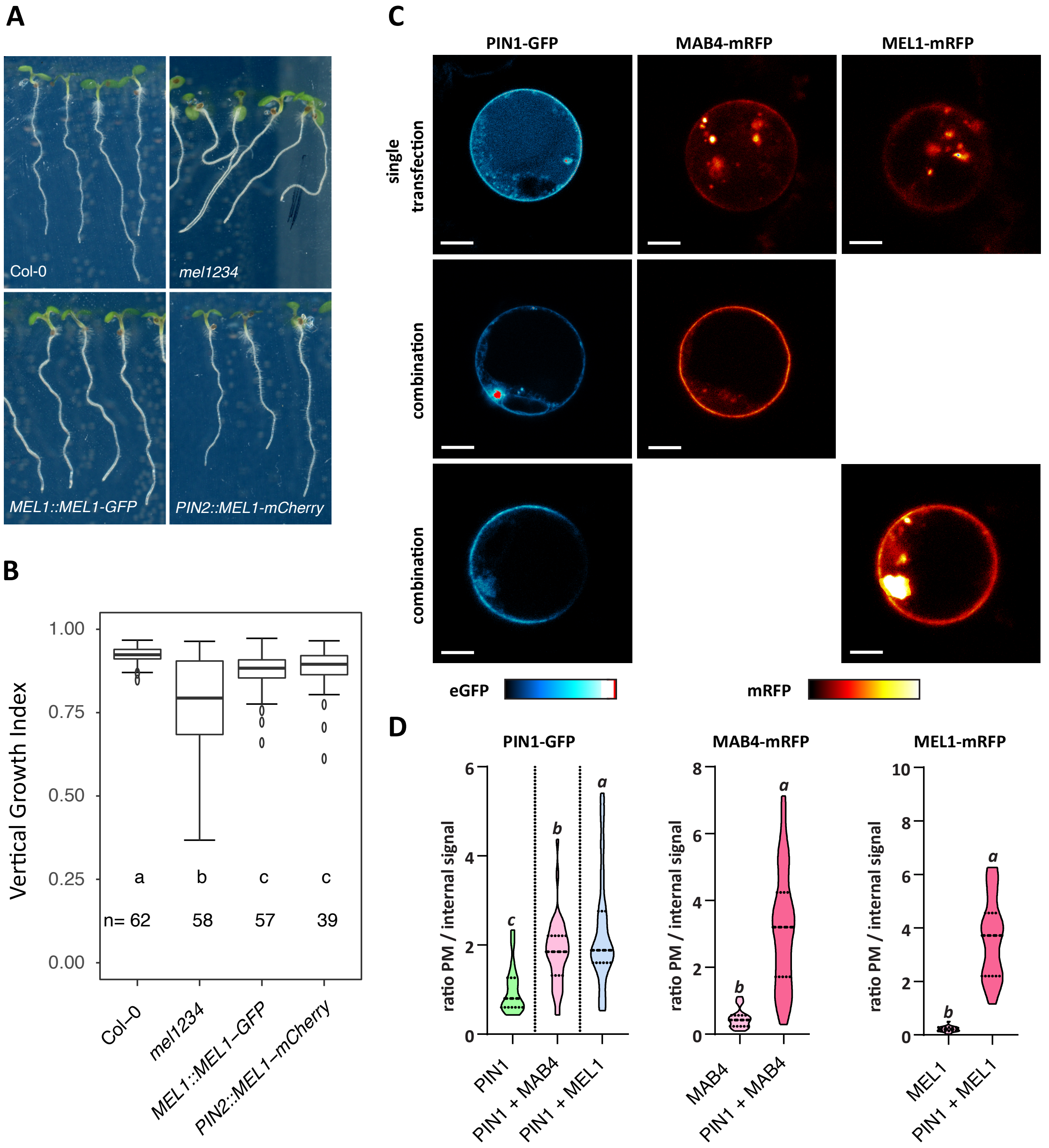

Figure S1: MEL1 reporters are functional; MAB4-mRFP and MEL1-mRFP are recruited to the PM by PIN1-GFP in protoplasts. Related to Figure 1.

(A) Phenotype of 5-day-old wild-type (Col-0), *mel1234, MEL1::MEL1-GFP/mel1234* or *PIN2::MEL1-mCherry/mel1234* seedlings.
(B) Vertical Growth Index of seedling roots shown in (B). n indicates the number of roots; letters indicate statistical differences (Wilcoxon test, p<0,01). the experiment was repeated independently 3 times with comparable results.
(C) Representative images of *Arabidopsis* cell culture protoplasts transfected with *p35S:PIN1-GFP, p35S:MAB4-mRFP* and *p35S:MEL1-mRFP*. Scale bar = 5µm.
(D) Quantification of the ratio between plasma membrane and intracellular GFP or mRFP signal of protoplasts shown in (C). n=18-38, statistical test is a one way ANOVA with post-hoc significance test. Letters denote statistically significant differences, p<0.05

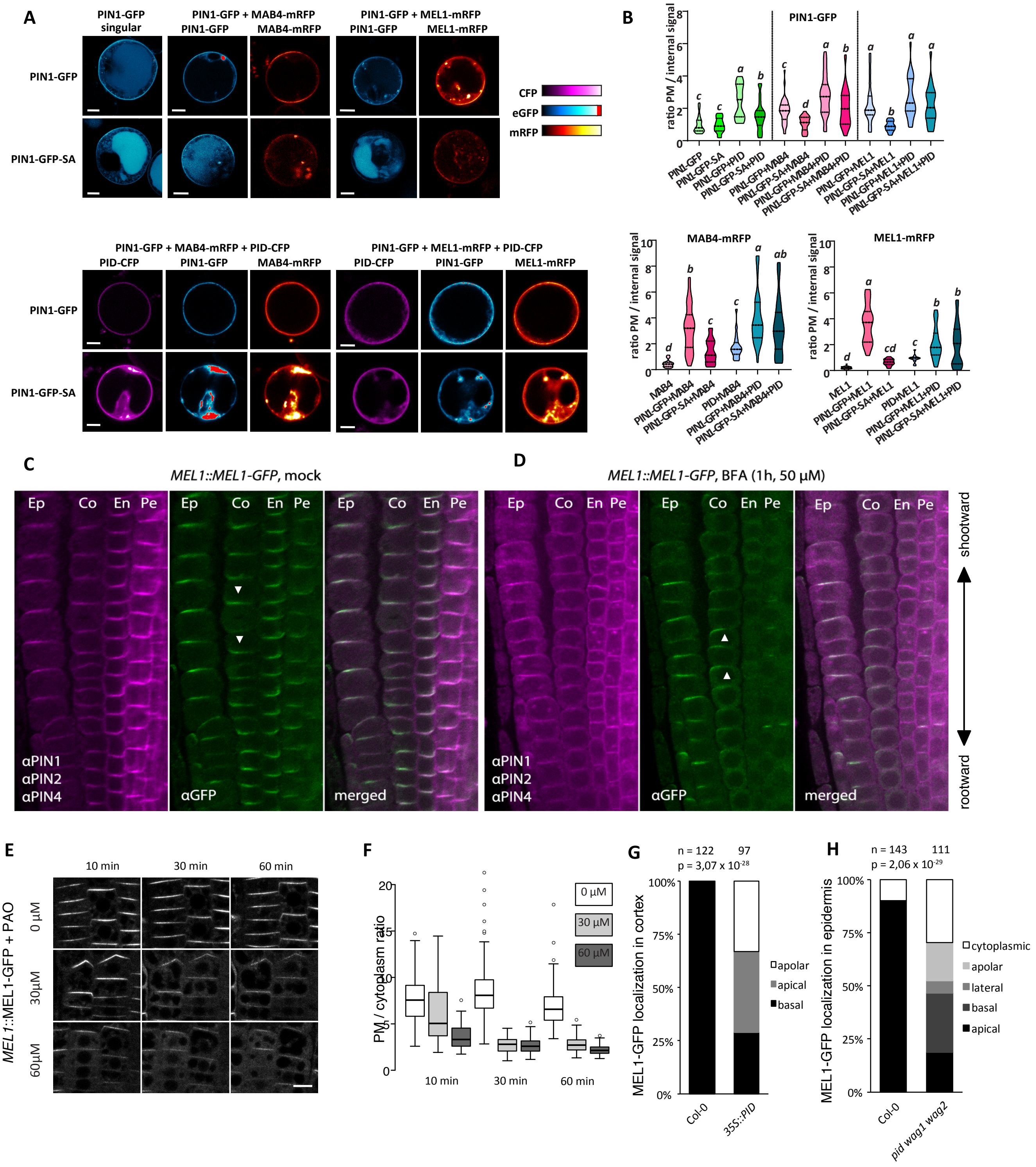

Figure S2: PINs recruit MEL1 to the PM in a phosphorylation-enhanced manner. Related to Figure 2.
(A) Representative images of *Arabidopsis* cell culture protoplasts transfected with *p35S:PIN1-GFP, p35S:PIN1-GFP-S1,2,3A* (SA), *p35S:MAB4-mRFP*, *p35S:MEL1-mRFP* and *p35S:PID-CFP*. Scale bar = 5µm.
(B) Quantification of the ratio between plasma membrane and intracellular GFP or mRFP signal of protoplasts shown in (A). n=13-38. The data in figure S1C, S1D, S2A and S2B are part of one experiment. Statistical test is a one way ANOVA with post-hoc significance test. Letters denote statistically significant differences, $p<0.05$.
(C) Immunofluorescence staining of PIN1, PIN2 and PIN4 (magenta) and MEL1-GFP (green) in 1 hour DMSO (mock)-treated *MEL1::MEL1-GFP* roots. MEL1-GFP colocalized with the PINs at the apical PM in the epidermis (Ep) and the basal PM in the cortex (Co), endodermis (En) and pericycle (Pe) cells. White arrowheads highlight the predominantly basal localization of MEL1-GFP in cortex cells.

(D) The same staining as in (C) of *MEL1::MEL1-GFP* roots treated for 1 hour with 50uM BFA. In addition to the PM, PIN signals can be observed in BFA bodies. MEL1-GFP signal mostly disappeared from the PM in endodermis and pericycle cells, while it is not affected in the epidermis. Notably, in cortex cells, MEL1-GFP often co-localized with PINs to the apical, instead of basal PM (highlighted by white arrowheads).
The images in (C) and (D) are representative of respectively 13 and 14 roots analyzed.
(E) MEL1-GFP dissociated from the PM upon treatment with the PI4-KINASE inhibitor phenylarsine oxide (PAO) in a time- and dose-dependent manner, similar to what has previously been observed for PID [S1]. Scale bar = 10 μm.
(F) Quantification of (E). The graph shows the ratios of PM/cytoplasm signal intensities. n = 60 cells from 6 different roots. The experiment was repeated independently three times with comparable results.
(G) Quantification of the data shown in Figure 2A. n refers to the number of cells from 5 representative roots, p-value was calculated with Pearson's Chi-squared test.
(H) Quantification of the data shown in Figure 2B. n refers to the number of cells from 5 representative roots, p-value was calculated with Pearson's Chi-squared test.

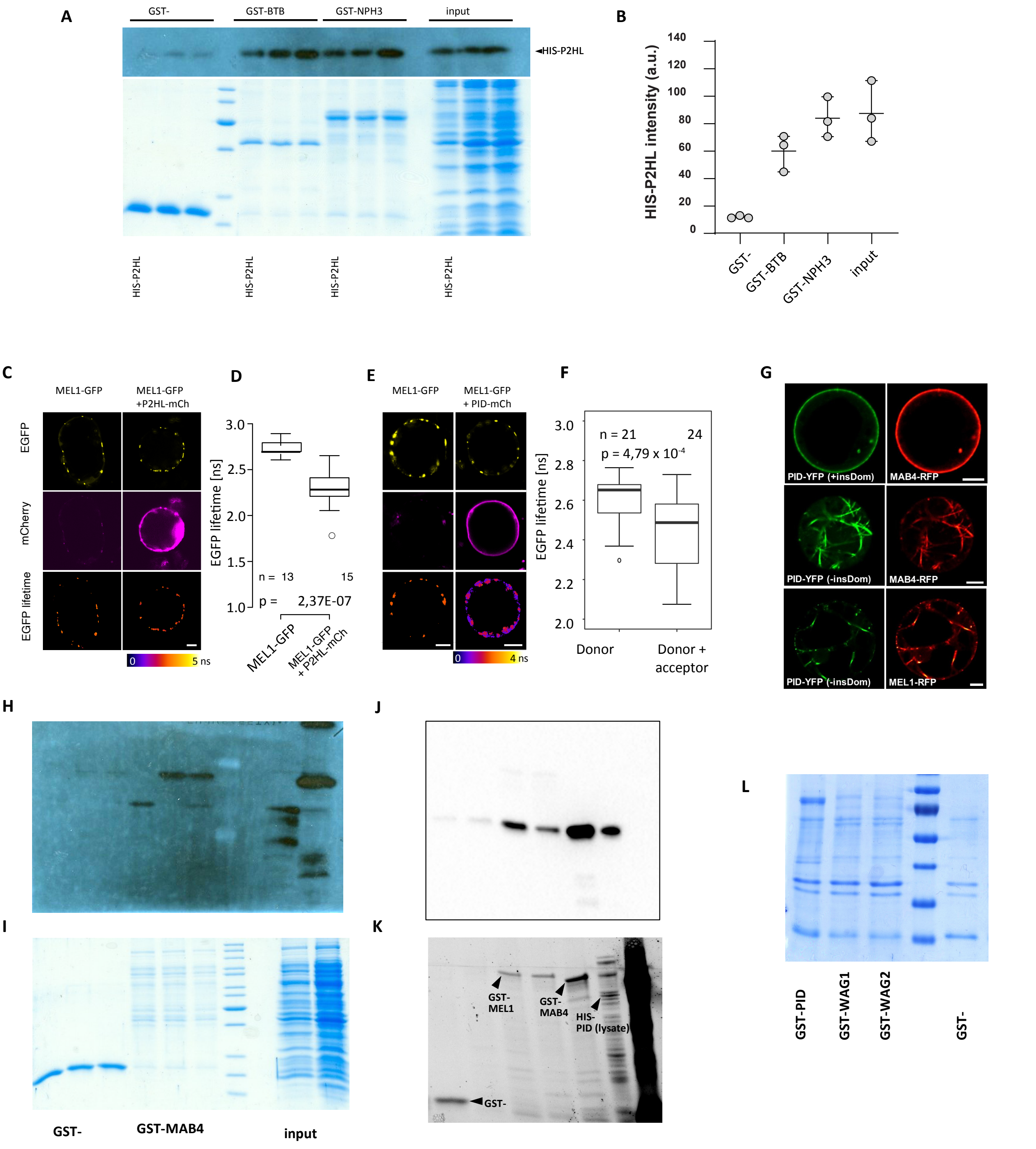

Figure S3: PINs, MAB4/MELs and PID/WAGs physically interact with each other. Related to Figure 3.
(A) *In vitro* pull-down assay of HIS-PIN2HL with GST, GST-BTB or GST-NPH3. The HIS-PIN2HL input controls are provided on the right. The blot and the corresponding Coomassie stain are representative of three independent experiments.
(B) Quantification of (A) and two independent additional experiments. Band intensities corrected for background intensity are shown.
(C) FLIM-FRET imaging of transiently transfected protoplasts expressing MEL1-GFP in the absence or presence of PIN2HL-mCherry.
(D) Quantitative analysis of (C). n indicates the total number of protoplasts from 2 independent experiments.
(E) FLIM-FRET imaging of transiently transfected protoplasts expressing MEL1-GFP in the absence or presence of PID-mCherry.
(F) Quantitative analysis of (E). n indicates the total number of protoplasts from 3 independent experiments.

(G) Protoplasts co-expressing PID-YFP (wildtype version with the insertion domain, + insDom) with MAB4-RFP, and PID-YFP (without the insertion domain, -insDom) with MAB4-RFP or MEL1-RFP. MAB4-RFP localized to the PM together with PID-YFP similarly to when it was expressed alone (Supplementary Figure 1a), while PID-YFP(- insDom) localized to microtubule-like structures, where it co-localized with both MAB4-RFP and MEL1-RFP. The images are representative of 8 protoplasts from 2 independent experiments per group.
(H) Full western blot, from which the image shown in Figure 3A has been excised.
(I) Coomassie gel of the western blot shown in Figures 3A and S3H
(J) Full western blot, from which the image shown in Figure 3C has been excised.
(K) TGX stain-free gel of the western blot shown in Figures 3C and S3J
(L) Coomassie gel of the western blot shown in Figure 3E.
Scale bars = 5 µm.

A

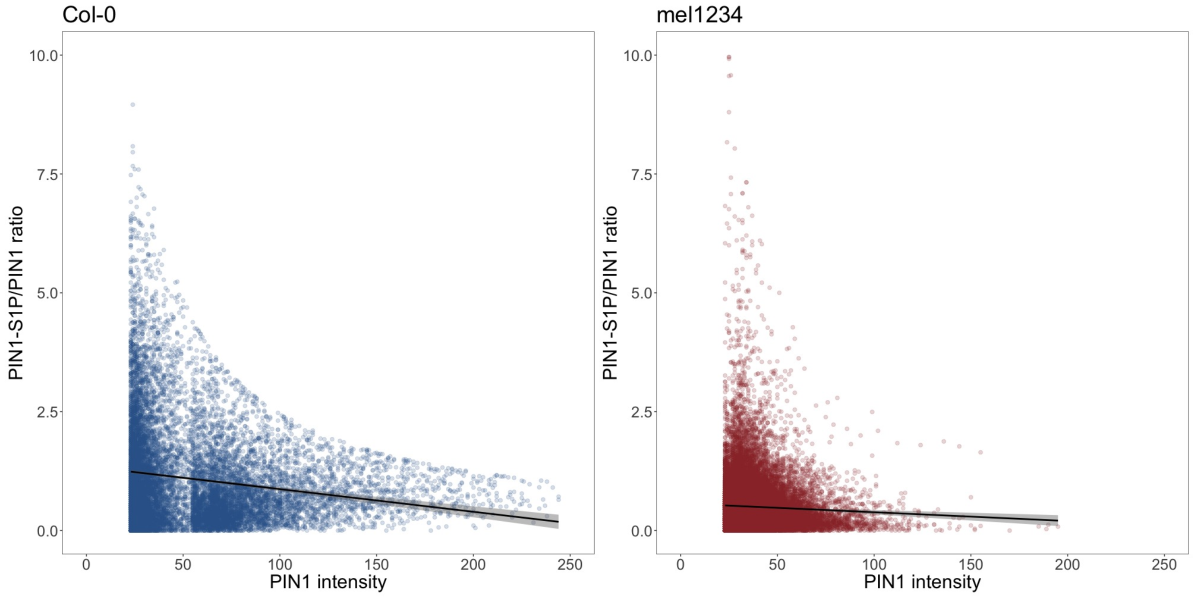

B

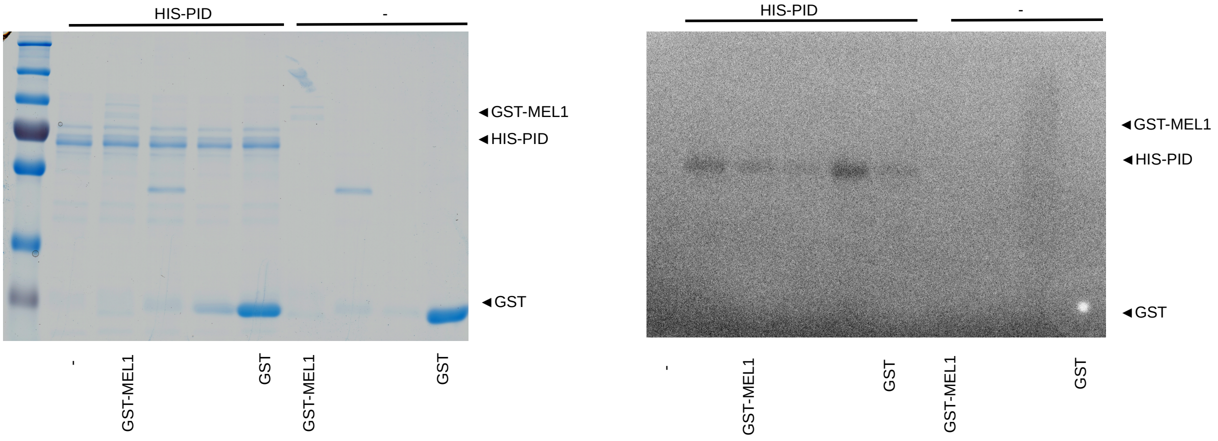

Figure S4: PIN1-S1P/PIN1 ratio does not depend on PIN1 intensity; PID does not phosphorylate MEL1 *in vitro.* Related to Figure 4.

(A) Dotplot representation of the relationship between PIN1 signal intensity and PIN1-S1P/PIN1 ratio in the data presented in in Figures 4C, 4D. The dots represent individual pixels included in the analysis, the black lines represent linear regression fit and the confidence interval is shaded in grey.

(B) In vitro ^{32}P phosphorylation assay with HIS-PID and GST-MEL1. The recombinant protein input is shown on the Coomassie stain on the left, and the radiograph on the right. Note the bands corresponding to HIS-PID autophosphorylation in the radiograph, which serve as an internal positive control of the assay.

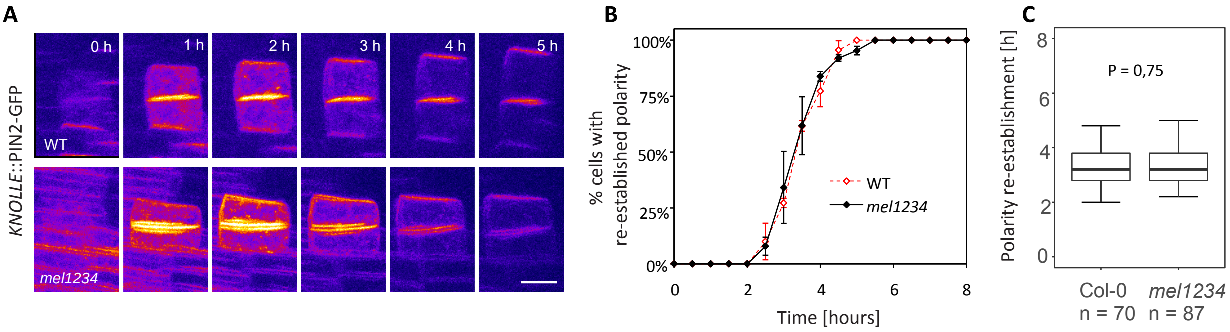

Figure S5: Apical polarity re-establishment of *KNOLLE*::PIN2-GFP is normal in the *mel1234* mutant. Related to Figure 5.

(A) Post-cytokinetic polarity re-establishment of PIN2-GFP expressed from the *KNOLLE* promoter was not obviously defective in the *mel1234* quadruple mutant compared to Col-0 (WT). Maximum intensity projections of a Z-stack are shown, scale bar = 10 µm

(B) Quantitative analysis of (A). Mean +/- SD of three independent experiments is shown, the total number of cells/roots analyzed was 70/7 (WT), resp. 87/8 (*mel1234*)

(C) A boxplot representation of data shown in A and B. For the purpose of statistical analysis, data from all three experiments were pooled.

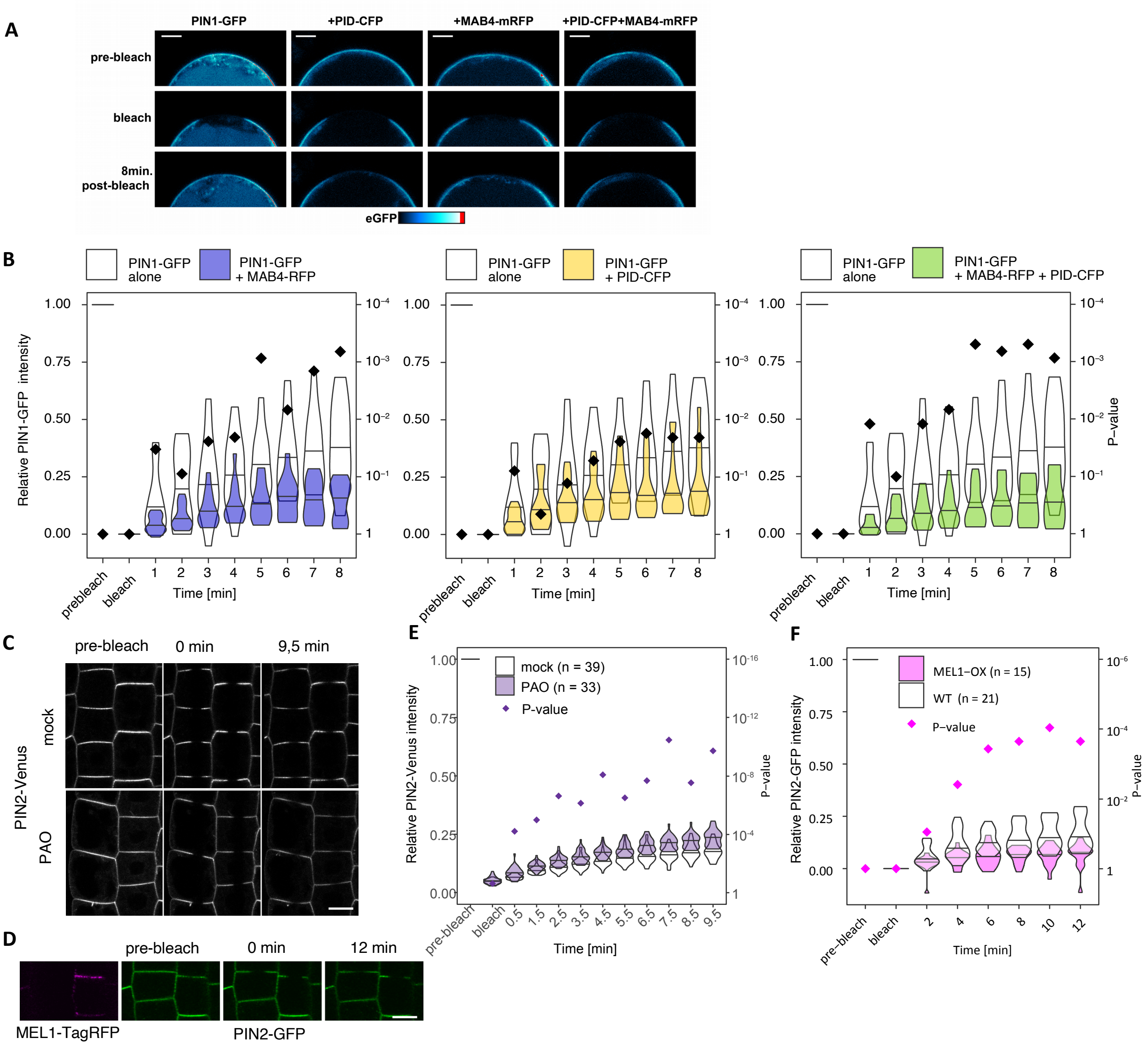

Figure S6: MAB4/MEL proteins an PID/WAG kinases reduce PIN lateral diffusion. Related to Figure 5.
(A) FRAP dynamics of PIN1-GFP expressed in protoplasts alone, in combination with PID-CFP, MAB4-RFP, or both. Scale bar = 5µm.
(B) Quantitative analysis of the data presented in (A). The violin plots show median values and probability density of the data. The PIN1-GFP control in all three graphs is the same. n = 12 protoplasts pooled from 2 independent experiments in each group
(C) FRAP dynamics of PIN2-Venus in epidermis cells of PIN2::PIN2-Venus roots treated with the solvent control (mock) or the PI4-KINASE inhibitor phenylarsine oxide (PAO) (60µM, 1h). The control is the same as in Figure 5A. Scale bar = 10 µm.
(D) FRAP dynamics of PIN2-GFP in epidermis cells of PIN2::PIN2-GFP, XVE>>MEL1-TagRFP roots induced with 1 µM ß-estradiol for 6 hrs, pretreated with 50 µM Cycloheximide and 1 µM ß-estradiol for 40 min and incubated with CHX and ß-estradiol throughout the experiments. Note the signals of MEL1-TagRFP, which enabled the comparison between WT and MEL1-TagRFP-overexpressing cells from the same roots. Scale bar = 10 µm.
(E) Quantitative analysis of (C). The violin plots show median values and probability density of the data after background subtraction and correction to photobleaching caused by iterative imaging. n refers to the number of cells from three different roots. The control is the same as in Figure 5B. The experiment was repeated independently twice with comparable results.
(F) Quantitative analysis of (D). The violin plots show median values and probability density of the data after background subtraction and correction to photobleaching caused by iterative imaging. n refers to the pooled number of cells from eight different roots analyzed in two independent experiments.

mel1-1_LP	GGAAGAACAAAACCACAAAGG
mel1-1_RP	ATGTAACCGCAAAAGCATCAG
mel2-1_LP	TCGGATTTACAAAGCAAGTGG
mel2-1_RP	CTCTTGCAGGATCCATAGCAG
mel3-1_LP	TGCAGGTTCCCAAACTTCTAC
mel3-1_RP	TTGATGCTTGCAAAACTGATG
mel4-1_LP	TCCAACTACGGAAGATGGTTG
mel4-1_RP	GCAGGAAAGATGCAACTTTTG
eir1-4_F	CAACGCGAAGAATGCTATGA
eir1-1_F	GGCAATTGCTTGATGTTGTTGATCATTTTATGGGACA
eir1-1/4_R	AAGCACCAAAGACTATAACTA
GABI-8409	ATATTGACCATCATACTCATTGC
LBb1.3	ATTTTGCCGATTTCGGAAC
PIN2HL_start_B1	GGGGACAAGTTTGTACAAAAAAGCAGGCTTCATGGCTAAGCTTCTCA TCTCCGA
PIN2HL_nostop_ B2	GGGGACCACTTTGTACAAGAAAGCTGGGTAACTCGCCGGCGGCATCT GCT
NPY1 S attB	GGGGACAAGTTTGTACAAAAAAGCAGGCTATGAAGTTCATGAAGCT AGG
NPY1 AS attB	GGGGACCACTTTGTACAAGAAAGCTGGGTACGATATCGAATGTCTGC GGC
NPY3+4 S attB	GGGGACAAGTTTGTACAAAAAAGCAGGCTATGAAGTTTATGAAACT TGG
NPY3 AS attB	GGGGACCACTTTGTACAAGAAAGCTGGGTAAGACGATGACCTTCTCC GGC
NPY4 AS attB	GGGGACCACTTTGTACAAGAAAGCTGGGTAAAACTCTTTCTCATGGT CCC
NPY5 S attB	GGGGACAAGTTTGTACAAAAAAGCAGGCTATGAAGTTCATGAAACT TGG
NPY5 AS attB	GGGGACCACTTTGTACAAGAAAGCTGGGTAATGCTTCTTGCTTGTGG AAG
PID Deletion Ins Dom S attB	CTCTGACTTTGACCTCGGGCGCGCCCTTAAGGCTGAACCGGTTACTG
PID Deletion InsDom AS attB	CAGTAACCGGTTCAGCCTTAAGGGCGCGCCCGAGGTCAAAGTCAGA G
pDONR207 F colpcr	TCGCGTTAACGCTAGCATGGATCTC
pDONR207 R colpcr	GTAACATCAGAGATTTTGAGACAC
NPY1 NPH3 domain Forward attB	GGGGACAAGTTTGTACAAAAAAGCAGGCTTTATGAAAGATTGGTGG GTCGAAG
NPY1 NPH3 domain Reverse attB	GGGGACCACTTTGTACAAGAAAGCTGGGTTTGATGCTTCATTTGTCA GTTTCC
NPY1 BTB domain Forward attB	GGGGACAAGTTTGTACAAAAAAGCAGGCTTCATGAAGCTAGGGTCT AAGCCCG
NPY1 BTB domain Reverse attB	GGGGACCACTTTGTACAAGAAAGCTGGGTTGCCTGAGTTGAGGAAA ACTTCGA

Table S1: Primers used in this study. Related to STAR Methods.

Supplemental References

S1. Simon, M.L.A., Platre, M.P., Marquès-Bueno, M.M., Armengot, L., Stanislas, T., Bayle, V., Caillaud, M.-C., and Jaillais, Y. (2016). A PtdIns(4)P-driven electrostatic field controls cell membrane identity and signalling in plants. Nat. Plants *2*, 16089.
